# Radiotherapy for brain metastasis and long-term survival

**DOI:** 10.1038/s41598-021-87357-x

**Published:** 2021-04-13

**Authors:** Kawngwoo Park, Gi Hwan Bae, Woo Kyung Kim, Chan-Jong Yoo, Cheol Wan Park, Soo-Ki Kim, Jihye Cha, Jin Wook Kim, Jaehun Jung

**Affiliations:** 1 Department of Neurosurgery, Gachon University Gil Medical Center, Incheon, 21565 Republic of Korea; 2 Department of Preventive Medicine, Gachon University College of Medicine, Incheon, 21565 Republic of Korea; 3 Department of Emergency Medicine, Gachon University Gil Medical Center, Incheon, 21565 Republic of Korea; 4 Department of Microbiology, Wonju College of Medicine, Yonsei University, Wonju, 26426 Republic of Korea; 5 Department of Radiation Oncology, Wonju College of Medicine, Yonsei University, Wonju, 26426 Republic of Korea; 6 Department of Neurosurgery, Seoul National University College of Medicine, Seoul, 03080 Republic of Korea; 7 Gil Artificial Intelligence and Bigdata Convergence Center, Gachon University Gil Medical Center, Incheon, 21565 Republic of Korea

**Keywords:** Medical research, Metastasis, Health policy, Radiotherapy

## Abstract

Patients with brain metastases (BM) can benefit from radiotherapy (RT), although the long-term benefits of RT remain unclear. We searched a Korean national health insurance claims database and identified 135,740 patients with newly diagnosed BM during 2002–2017. Propensity score matching (PSM) was used to evaluate survival according to RT modality, which included whole-brain radiotherapy (WBRT) and/or stereotactic radiosurgery (SRS). The 84,986 eligible patients were followed for a median interval of 6.6 months, and 37,046 patients underwent RT (43.6%). After the PSM, patients who underwent RT had significantly better overall survival after 1 year (42.4% vs. 35.3%, P < 0.001), although there was no significant difference at 2.6 years, and patients who did not undergo RT had better survival after 5 years. Among patients with BM from lung cancer, RT was also associated with a survival difference after 1 year (57.3% vs. 32.8%, P < 0.001) and a median survival increase of 3.7 months. The 1-year overall survival rate was significantly better for SRS than for WBRT (46.4% vs. 38.8%, P < 0.001). Among Korean patients with BM, especially patients with primary lung cancer, RT improved the short-term survival rate, and SRS appears to be more useful than WBRT in this setting.

## Introduction

Whole-brain radiotherapy (WBRT) has been accepted as a palliative treatment for brain metastasis (BM)^1^, based on its ability to control neurologic symptoms and reduce disease burden in several clinical trials from the 1980s^2,3^. Stereotactic radiosurgery (SRS) has also recently been found to improve the local control of BM^4–6^. While these studies have indicated that radiotherapy (RT) provides a survival benefit for patients with BM, we are not aware of any real-world studies regarding the benefits of RT in this setting. Furthermore, the QUARTZ trial revealed that WBRT provided limited benefits, relative to best supportive care alone, for patients with BM from non-small cell lung cancer^7^. Moreover, another study revealed that poor overall survival after WBRT was associated with poor performance status, older age, > 3 intracranial metastases, and uncontrolled primary tumors^8^. Based on these conflicting findings, questions have emerged regarding the benefits of RT for BM that were determined based on previous clinical studies. Given the lack of long-term real-world analyses of the benefits of RT in this setting, we aimed to compare the long-term results of WBRT alone, SRS alone, and WBRT plus SRS for BM.

The National Healthcare Insurance Service (NHIS) of South Korea delivers a government-controlled, single-payer, and obligatory insurance plan that covers almost the entire Korean population (approximately 50 million residents)^9^. This database contains accumulated claims-based information regarding the patients’ diagnosis, drug treatment, other treatment, imaging use, and death. Thus, we used the NHIS database to evaluate the effects of RT on long-term survival among BM patients in Korea.

## Methods

### Data source and study design

This population-based retrospective study evaluated the effects of RT on overall survival among BM patients. The primary outcome was overall survival during the follow-up period after newly diagnosed BM. The primary variable of interest was RT use, and the secondary variables of interest were the use of specific RT modalities (i.e., SRS and/or WBRT). Korea’s treatment strategy for brain metastasis was implemented in accordance with NCCN guidelines. Based on the NCCN guidelines, WBRT was considered for patients with a large number of BM, while SRS was applied for patients with limited BM. The NHIS database was searched for 20–79-year-old patients with newly diagnosed BM from January 1, 2003 to December 31, 2016, using the BM diagnostic code (C793) from the Korean Standard Classification of Disease Version 6, which based on the 10th International Classification of Disease^9^. Patients were excluded if they received < 5 sessions of WBRT, given the incompleteness of the treatment for BM. Furthermore, patients were only considered eligible if they had BM that was derived from a solid primary tumor, and we excluded patients with primary brain tumors or hematological malignancies, such as lymphoma. The NHIS records contain information regarding sex, age, diagnosis, and treatment, which are maintained in accordance with the Act on the Protection of Personal Information Maintained by Public Agencies. Propensity score matching (PSM, 1:1) was performed according to sex, age group, medical aid beneficiary status, medical facility classification, primary tumor location, surgery use, chemotherapy use, diagnostic year, and CCI.

**Figure 1:**
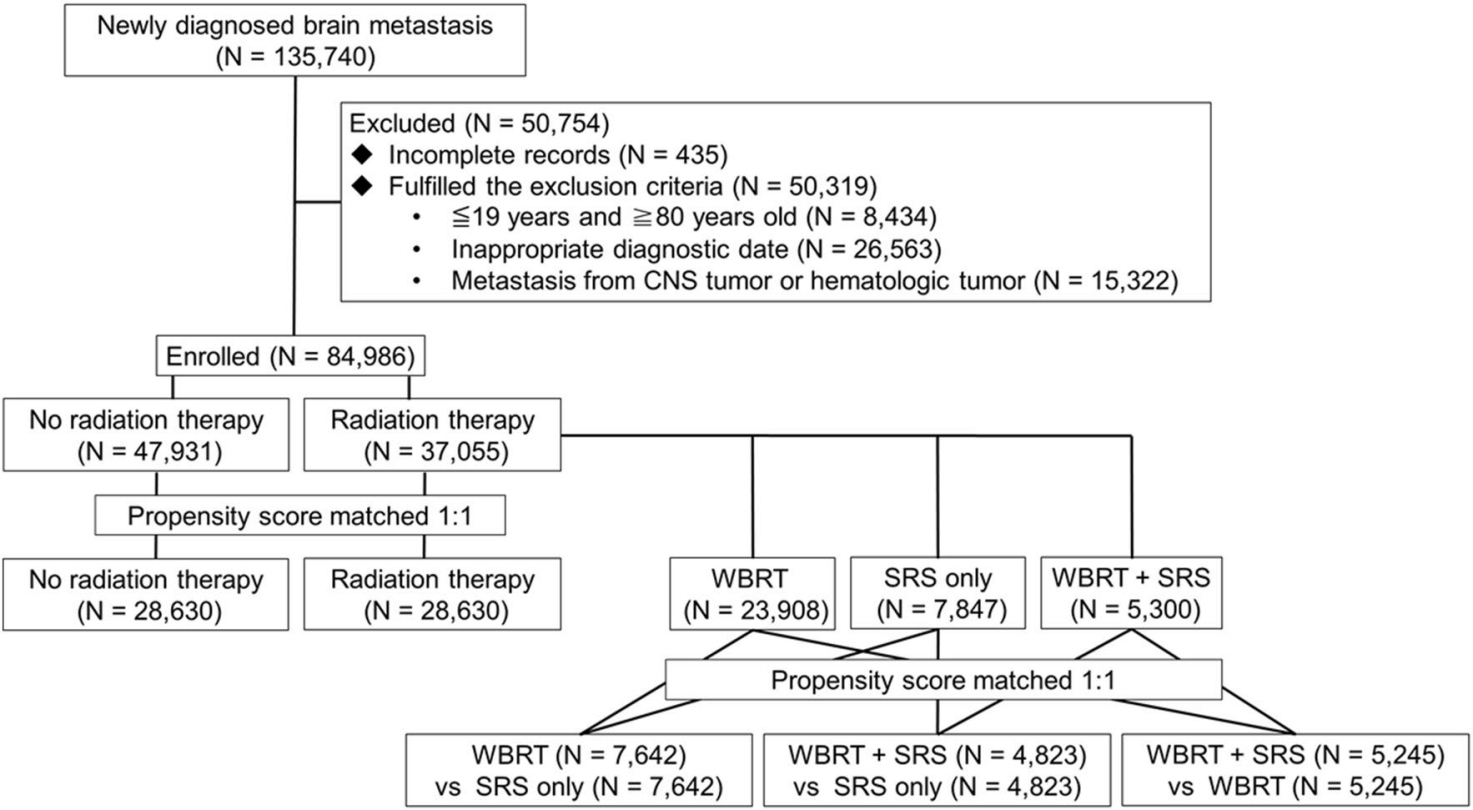
Study flowchart. *CNS* central nervous system, *WBRT* whole-brain radiotherapy, *SRS* stereotactic radiosurgery. Created using R software (version 3.5.2; R Foundation for Statistical Computing, Vienna, Austria).

Data analysis was conducted through four PSM in total, as shown in Fig. 1. Important variables that were not matched after PSM were analyzed through Cox regression. This kept important variables, such as age, gender, and Charlson Comorbidity Index (CCI). The outcomes from WBRT alone, SRS alone, and WBRT + SRS were also compared after the PSM. Furthermore, we analyzed the overall survival outcomes for the five most common primary tumor sites in Korea (lung, breast, liver, colorectum, and stomach).

### Ethical approval statement

The study was reviewed and approved by the Institutional Review Board of Gachon University Gil Medical Center (approval number: GFIRB2019-207), and the requirement to obtain written consent was waived due to the retrospective nature of this study; patients and the public were not involved in the study design, data collection, analysis, or interpretation of data. All study methods were carried out based on the Declaration of Helsinki.

### Identification of RT

The use of RT was identified using the insurance claim codes for WBRT and SRS, which included gamma knife radiosurgery, CyberKnife treatment, and linear accelerator-based radiosurgery (e.g., using the Novalis system). At least five sessions after the diagnosis of BM were required for WBRT, which was identified using the related treatment codes (HD061, HD051, HD052, HD053, HD055, and HD056). The SRS procedures were categorized as gamma knife radiosurgery (HD113), CyberKnife treatment (HD114), or linear accelerator-based radiosurgery (HD115).

### Covariates

The NHIS database was also used to collect information regarding the 19 major non-psychiatric comorbidities in the Charlson Comorbidity Index (CCI) (Supplemental Table 1)^10^. The PSM considered the following factors that are associated with overall survival among BM patients: surgical resection, chemotherapy, and primary tumor location. Surgical resection was defined as craniotomy with tumor removal but did not include brain biopsy, which was identified based on the corresponding treatment codes (N0335, S4634, S4635, S4636, and S4637). Treatment codes for the various chemotherapy regimens are listed in Supplemental Table 2.

### Statistical analysis

Overall survival was evaluated according to any use of RT and the various RT modalities (WBRT alone, SRS alone, and WBRT + SRS). Categorical variables were expressed as number (%), and continuous variables were expressed as mean ± standard deviation. Overall survival was evaluated using the Kaplan–Meier method and log-rank test. Prognostic variables were evaluated using a Cox proportional hazards model, which was adjusted for age, sex, CCI, chemotherapy use, surgery use, diagnostic year, and primary tumor location. The PSM was based on factors that likely influenced overall survival, and the PSM scores were calculated using multiple logistic regression. The variables included sex, age group, medical aid beneficiary status, medical facility classification, primary tumor location, surgery use, chemotherapy use, diagnostic year, and CCI. The PSM was performed using a caliper width of 0.2 of the standard deviation of the logit of the propensity score. Subgroup analyses were performed according to primary tumor location, given that the prognosis varies according to tumor type.

All analyses were performed using SAS software (version 9.4; SAS Institute Inc., Cray, NC, USA) and R software (version 3.5.2; R Foundation for Statistical Computing, Vienna, Austria). Dr. JHJ had full access to all study data and takes responsibility for the integrity of the data and the accuracy of the data analysis.

## Results

### Patient characteristics

The NHIS database included 135,740 patients with newly diagnosed BM between January 1, 2005 and December 31, 2016. However, we excluded patients who were diagnosed after January 2017 (insufficient follow-up period), patients who were diagnosed before December 2004 (incomplete records), and patients with primary brain tumors and hematological malignancies (Fig. 1). Thus, the analyses included 84,986 patients with a median follow-up duration of 6.6 ± 30.2 months after the diagnosis of BM. The median survival of the entire patient’s population was 6.03 (IQR 1.83–18.8 months). The patients’ baseline characteristics are summarized in Table 1. Among the 84,986 patients, 37,046 patients (43.6%) received RT after being diagnosed with BM. The median follow-up periods were 4.1 ± 33.4 months for the non-RT group (interquartile range [IQR] 1.8–19.3 months) and 9.1 ± 25.4 months for the RT group (IQR 3.1–22.4 months). After the PSM, the median follow-up periods were 6.6 ± 30.6 months for the non-RT group (IQR 2.9–20.9 months) and 9.3 ± 26.6 months for the RT group (IQR 3.5–22.3 months). Significant intergroup differences were observed in age, sex, chemotherapy use, and surgery use (Table 1).

**Table 1:** Comparing the patients’ baseline characteristics according to radiotherapy status.

Variables	Before PSM			After PSM		
	Non-RT	RT	P-value	Non-RT	RT	P-value
	47,940 (100%)	37,046 (100%)		28,630 (100%)	28,630 (100%)	
**Sex**			< .0001			< .0001
Male	29,163 (60.83%)	20,096 (54.25%)		17,236 (60.20%)	16,559 (57.84%)	
Female	18,777 (39.17%)	16,950 (45.75%)		11,394 (39.80%)	12,071 (42.16%)	
				28,630	28,630	
**Age, mean ± SD (year)**	63.48 ± 11.20	59.35 ± 11.13	< .0001	61.83 ± 10.95	60.61 ± 10.96	< .0001
20 ~ 29	257 (0.54%)	198 (0.53%)		122 (0.43%)	140 (0.49%)	
30 ~ 39	1271 (2.65%)	1660 (4.48%)		835 (2.92%)	1032 (3.60%)	
40 ~ 49	4407 (9.19%)	5270 (14.23%)		3104 (10.84%)	3529 (12.33%)	
50 ~ 59	9860 (20.57%)	10,788 (29.12%)		7147 (24.96%)	7589 (26.51%)	
60 ~ 69	14,958 (31.20%)	11,544 (31.16%)		9650 (33.71%)	9461 (33.05%)	
70 ~	17,187 (35.85%)	7586 (20.48%)		7772 (27.15%)	6879 (24.03%)	
**Medical aid beneficiary**^a^	4177 (8.71%)	2127 (5.74%)	< .0001	1857 (6.49%)	1854 (6.48%)	0.9594
Medial facility^b^			< .0001			< .0001
Tertiary hospital	15,359 (32.04%)	17,343 (46.81%)		11,133 (38.89%)	12,173 (42.52%)	
General hospital	4800 (10.01%)	3016 (8.14%)		2608 (9.11%)	2498 (8.73%)	
Hospital	27,747 (57.88%)	16,682 (45.03%)		14,887 (52.00%)	13,954 (48.74%)	
Clinic	34 (0.071%)	5 (0.01%)		2 (0.01%)	5 (0.02%)	
**Primary cancer**						
Head and neck	834 (1.74%)	755 (2.04%)	0.0015	675 (2.36%)	682 (2.38%)	0.8475
Esophagus	505 (1.05%)	517 (1.40%)	< .0001	432 (1.51%)	438 (1.53%)	0.8376
Stomach	3648 (7.61%)	1083 (2.92%)	< .0001	970 (3.39%)	1064 (3.72%)	0.0338
Colorectal	3784 (7.89%)	3563 (9.62%)	< .0001	2875 (10.04%)	3108 (10.86%)	0.0015
Liver	2774 (5.79%)	1986 (5.36%)	0.0075	1783 (6.23%)	1805 (6.30%)	0.7044
Hepatobiliary	693 (1.45%)	314 (0.85%)	< .0001	317 (1.11%)	306 (1.07%)	0.6577
Pancreas	1222 (2.55%)	569 (1.54%)	< .0001	494 (1.73%)	534 (1.87%)	0.2081
Pharynx	203 (0.42%)	232 (0.63%)	< .0001	180 (0.63%)	194 (0.68%)	0.4677
Lung	24,170 (50.42%)	21,197 (57.22%)	< .0001	18,481 (64.55%)	17,760 (62.03%)	< .0001
Breast	3355 (7.00%)	6463 (17.45%)	< .0001	3248 (11.34%)	3898 (13.62%)	< .0001
Cervix	308 (0.64%)	615 (1.66%)	< .0001	298 (1.04%)	422 (1.47%)	< .0001
Uterine	153 (0.32%)	235 (0.63%)	< .0001	139 (0.49%)	186 (0.65%)	0.0089
Ovary	676 (1.41%)	461 (1.24%)	0.0371	394 (1.38%)	421 (1.47%)	0.3408
Prostate	1551 (3.24%)	1011 (2.73%)	< .0001	1036 (3.62%)	951 (3.32%)	0.0523
Scrotum	59 (0.12%)	35 (0.09%)	0.2137	33 (0.12%)	32 (0.11%)	0.9012
Kidney	920 (1.92%)	886 (2.39%)	< .0001	696 (2.43%)	768 (2.68%)	0.0566
Bladder	543 (1.13%)	322 (0.87%)	0.0001	310 (1.08%)	300 (1.05%)	0.684
Thyroid	972 (2.03%)	752 (2.03%)	0.9806	582 (2.03%)	660 (2.31%)	0.0252
**Operation**	1208 (2.52%)	2571 (6.94%)	< .0001	1091 (3.81%)	1641 (5.73%)	< .0001
**Chemotherapy**	16,871 (35.19%)	21,323 (57.56%)	< .0001	13,731 (47.96%)	14,798 (51.69%)	< .0001
**Diagnostic year**			< .0001			< .0001
2005	3896 (8.13%)	1804 (4.87%)		1732 (6.05%)	1564 (5.46%)	
2006	4825 (10.06%)	2191 (5.91%)		2007 (7.01%)	1901 (6.64%)	
2007	4837 (10.09%)	2297 (6.20%)		2146 (7.50%)	2043 (7.14%)	
2008	4226 (8.82%)	2476 (6.68%)		2197 (7.67%)	2045 (7.14%)	
2009	4090 (8.53%)	2754 (7.43%)		2323 (8.11%)	2289 (8.00%)	
2010	4210 (8.78%)	3088 (8.34%)		2532 (8.84%)	2436 (8.51%)	
2011	3691 (7.70%)	3280 (8.85%)		2453 (8.57%)	2587 (9.04%)	
2012	3427 (7.15%)	3552 (9.59%)		2474 (8.64%)	2461 (8.60%)	
2013	3568 (7.44%)	3859 (10.42%)		2656 (9.28%)	2616 (9.14%)	
2014	3550 (7.41%)	4005 (10.81%)		2573 (8.99%)	2871 (10.03%)	
2015	3566 (7.44%)	4010 (10.82%)		2683 (9.37%)	2810 (9.81%)	
2016	4054 (8.46%)	3730 (10.07%)		2854 (9.97%)	3007 (10.50%)	
CCI	4.53 ± 2.41	4.92 ± 2.10	< .0001	4.86 ± 2.34	4.91 ± 2.14	< .0001

*Non-RT* Non-radio therapy**,** *RT* radio therapy, *PSM* propensity score matching, *CCI* Charlson Comorbidity Index.

^a^Recipients received in the form of public assistance to guarantee the minimum standard of healthcare insurance in Korea.

^b^Four different classification of hospitals in Korea^25^.

### Survival according to RT use

**Figure 2:**
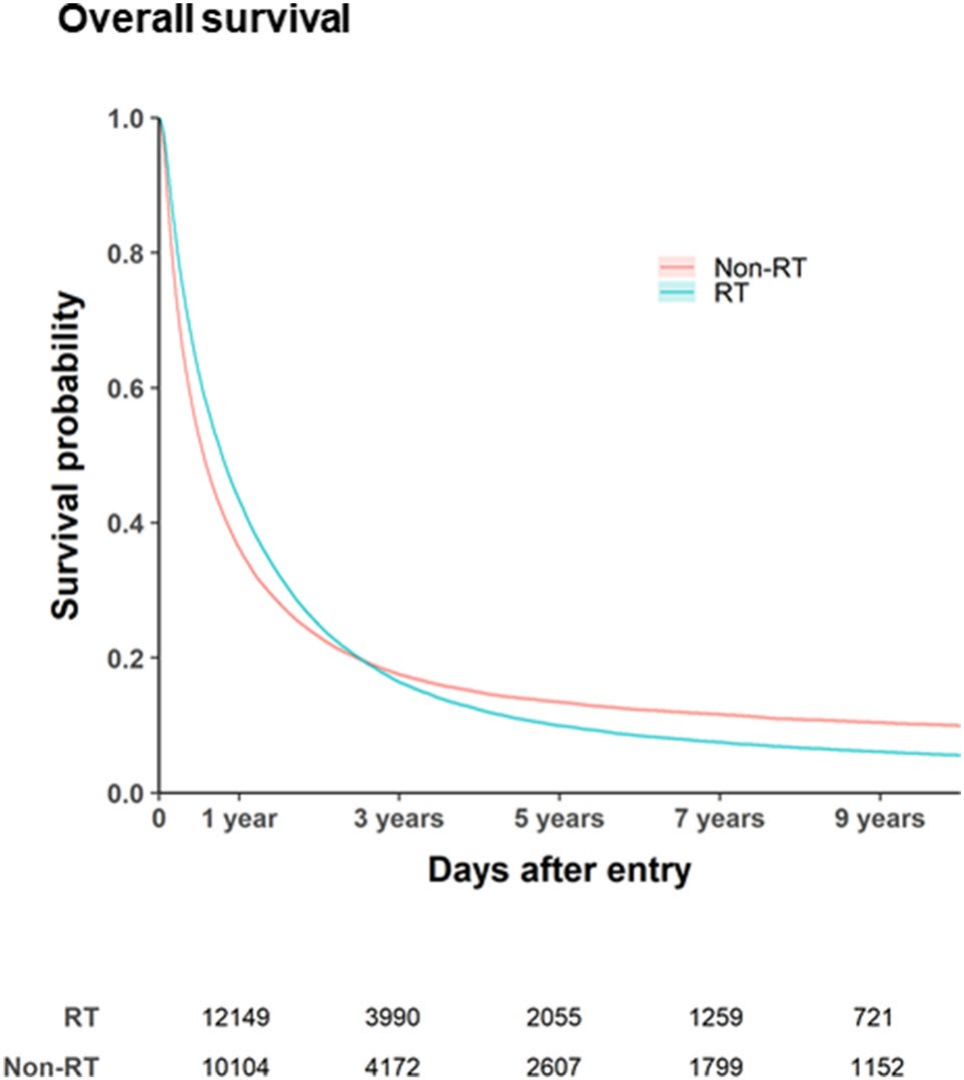
Kaplan–Meier survival curves according to radiotherapy (RT) status. Outcomes are shown as overall survival among all patients. *Non-RT* Non- radiotherapy, *RT* radiotherapy. Created using SAS software (version 9.4; SAS Institute Inc., Cray, NC, USA).

After the PSM, univariate Cox regression analysis revealed that the crude hazard ratio (HR) for death in the RT group, relative to the non-RT group, was 0.95 (95% confidence interval [CI] 0.93–0.96; P < 0.0001). Furthermore, RT was an independent predictor of overall survival in the multivariate Cox regression analysis (adjusted HR: 0.96, 95% CI 0.95–0.98; P < 0.0001) (Supplemental Table 3). In this table, we presented HR within subgroups by Cox regressions. The non-RT group had overall survival rates of 35.3% at 1 year, 14.6% at 3 years, 9.1% at 5 years, and 6.3% at 7 years, while the RT group had overall survival rates of 42.4% at 1 year, 13.9% at 3 years, 7.2% at 5 years, and 4.4% at 7 years (Fig. 2). The median survival difference for RT was 2.7 months, based on median survival intervals of 9.3 months in the RT group (95% CI 9.10–9.47 months, IQR 3.5–22.3 months) and 6.6 months in the non-RT group (95% CI 6.4–6.7 months, IQR 2.4–19.4 months) (Fig. 2). The survival curves for the two groups intersected at 2.57 years, and better survival after that point was observed in the non-RT group (Fig. 2).

To overcome selection bias that could occur by excluding the patients with less than five fractions of RT, the analysis was conducted on all patients who received RT at least once, as shown in Supplemental Table 4. In the intention-to-treat analysis, our results failed to confirm the favorable outcomes of RT patients for BMs. The HR of patients who received RT compared to those who did not receive RT for BM was 1.33 (95% CI 1.31–1.36, *p* < 0.0001).

### Survival according to primary tumor location

**Figure 3:**
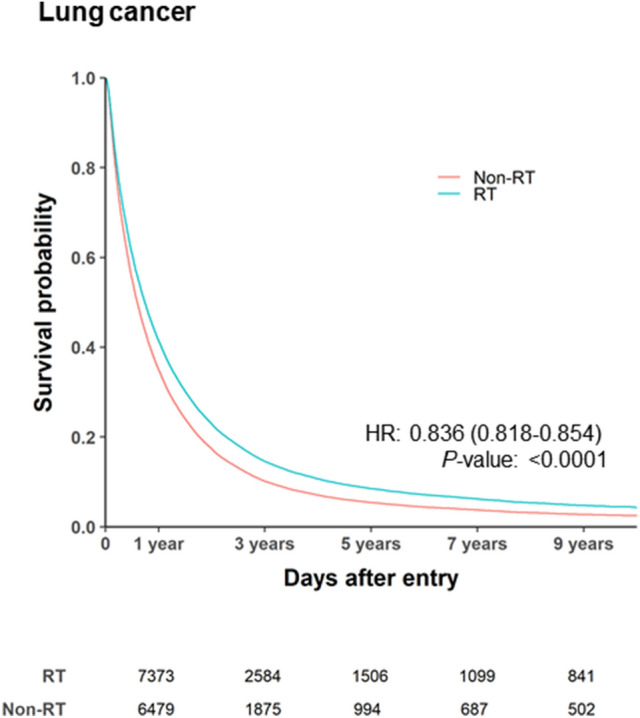
Cox regression survival analyses for lung cancer. Cox proportional hazards model adjusted for age, sex, CCI, chemotherapy use, surgery use, diagnostic year, and primary tumor location. *HR* hazard ratio, *Non-RT* Non-radiotherapy, *RT* radiotherapy. Created using SAS software (version 9.4; SAS Institute Inc., Cray, NC, USA).

Most BM patients had primary lung cancer, and overall survival after the diagnosis of BM varied according to the primary tumor location. Among patients with primary lung cancer, the median survival difference for RT was 3.7 months, based on median survival intervals of 10.0 months in the RT group (95% CI 9.77–10.27 months, IQR 4.0–21.4 months) and 6.3 months in the non-RT group (95% CI 6.1–6.4 months, IQR 2.5–15.7 months) (Fig. 3). However, RT was not associated with a survival difference for other primary tumors, such as breast, liver, colorectal, and stomach cancer. (Supplemental Fig. 1).

### Survival according to RT modality

**Figure 4:**
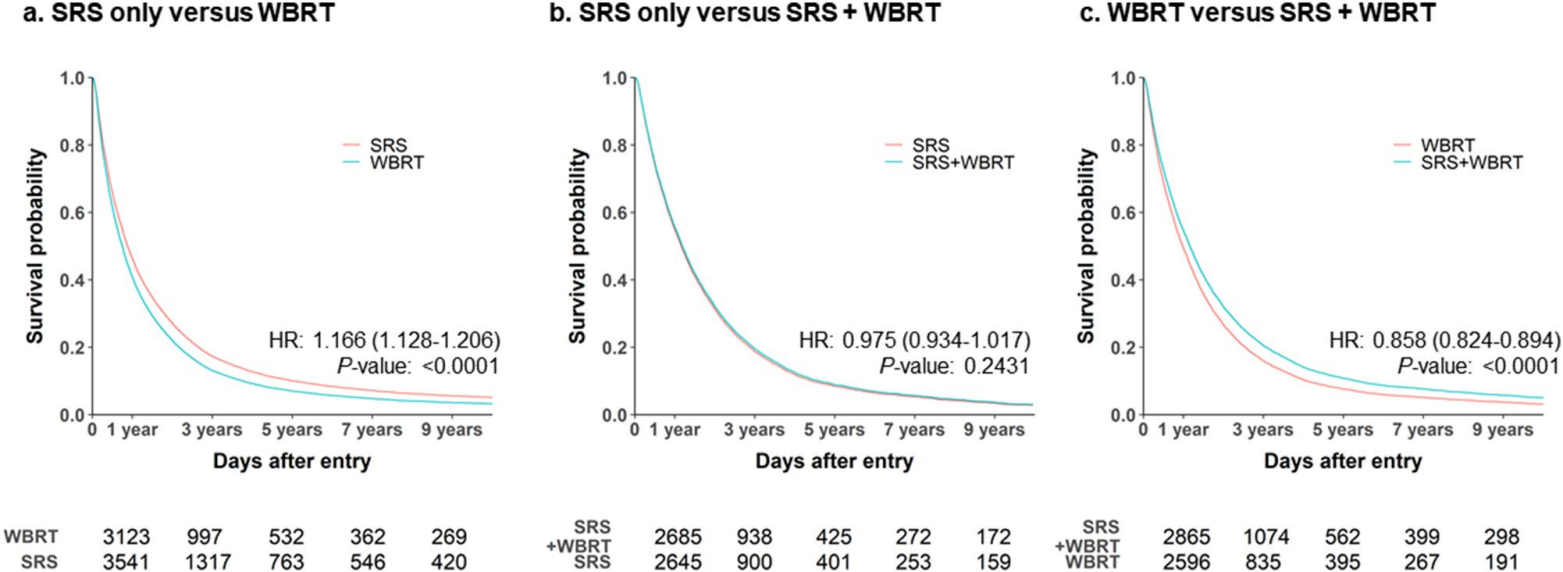
Cox regression survival analyses for SRS only vs. WBRT (**a**), SRS only vs. SRS + WBRT (**b**), and WBRT vs. SRS + WBRT (**c**). Cox proportional hazards model adjusted for age, sex, CCI, chemotherapy use, surgery use, diagnostic year, and primary tumor location. *WBRT* whole-brain radiotherapy, *SRS* stereotactic radiosurgery, *HR* hazard ratio. Created using SAS software (version 9.4; SAS Institute Inc., Cray, NC, USA).

The RT modalities included WBRT alone (23,908 patients, 64.5%), SRS alone (7847 patients, 21.2%), and WBRT plus SRS (5300 patients, 14.3%). Supplemental Table 5 shows that these subgroups had well-balanced baseline characteristics after the PSM. The median survival intervals were 10.9 months in the SRS group (95% CI 10.2–11.1 months, IQR 4.5–24.2 months) and 8.4 months in the WBRT group (95% CI 7.6–9.3 months, IQR 3.3–19.7 months; P < 0.001) (Fig. 4a). Although not statistically significant, SRS plus WBRT had a median survival difference of 6.3 months, relative to WBRT alone, based on the median survival intervals of 15.6 months for SRS plus WBRT (95% CI 14.7–16.2 months, IQR 7.0–28.9 months) and 9.3 months for WBRT alone (95% CI 8.1–9.9 months, IQR 3.6–22.3 months) (Fig. 4b,c).

### Intention-to-treat analysis

We conducted further analysis of patients who received RT at least once. To this end, we divided and reanalyzed patients with RT more than once, patients with one to four fractions RT, and patients with more than five fractions RT (Supplemental Table 4, 6, 7).

## Discussion

This retrospective population-based study revealed that RT was associated with a survival difference among Korean patients with BM, especially among patients with BM from lung cancer. Interestingly, while RT was associated with a survival difference after 1 year, the difference disappeared after 2.57 years. In addition, we found that SRS was associated with better overall survival, relative to WBRT, among patients with BM.

Several studies have indicated that WBRT for BM can alleviate 63–85% of neurologic symptoms and increase overall survival by < 6 months^1,11–14^. The American Society of Radiation Oncology guidelines recommend managing BM based on the estimated prognosis from the histopathological findings^15^. Furthermore, among patients with a life expectancy of > 3 months, the number, size, and location of the BM are important factors. Patients with a good prognosis may undergo SRS, WBRT, and/or surgical resection, while best supportive care with or without WBRT has been recommended for patients with a life expectancy of < 3 months^16,17^. However, the utility of RT has been questioned, as WBRT can lower the quality of life for BM patients who have a poor performance status and older age^7^. Therefore, WBRT might be omitted for select patients with asymptomatic BM and a poor prognosis^7,18^.

Our results indicate that RT did not significantly increase the median overall survival of BM patients, although RT was associated with a survival difference after 1 year, which subsequently reversed after approximately 2.6 years. These findings imply that RT may provide a short-term survival difference of approximately 2.7 months for BM patients, although we did not evaluate differences in survival according to life expectancy. We also observed that RT was associated with a survival difference of 3.7 months among patients with BM from lung cancer, although no meaningful benefit was observed for other primary tumor locations, which may help explain why RT was associated with poorer overall survival after 2.6 years. It is also possible that the improved outcomes in non-RT cases at > 2.6 years were related to the development of new systemic and targeted therapies, which may play important roles in the future treatment of BM. Nevertheless, the blood–brain barrier is an obstacle to effective chemotherapies for BM, which suggests that RT will continue to play a role in the management of BM patients. Additionally, the reserve pattern of the survival curve of the non-RT group after 2.6 years is assumed to be due to selection bias for patients receiving RT and uncertainly long-term RT side effects.

Although BM occurs in up to 40% of patients with metastatic cancer, the survival outcomes vary according to the primary tumor location^11,19^. Thus, we performed subgroup analyses for the five most common primary tumor locations in Korea: lung cancer, breast cancer, liver cancer, colorectal cancer, and stomach cancer. The results revealed that RT was associated with a survival benefit among patients with BM from lung cancer, but not among patients with BM from the other primary tumor locations.

Several randomized controlled trials have confirmed that WBRT is an important adjuvant treatment after surgical resection and SRS^20–22^. However, those studies only evaluated RT within combination treatments and did not confirm whether RT alone offered a survival benefit. In addition, there is limited research regarding long-term survival in this setting, which is related to the poor prognosis of BM patients. The present study evaluated long-term survival outcomes among a population-based cohort of Korean patients with BM, which revealed that SRS might provide better survival than WBRT. In this context, SRS can be used alone as definitive treatment for patients with a limited number of BMs, in combination with WBRT, or as a perioperative intervention. Several reports have indicated that SRS improves overall survival and local control for BM patients^4,23,24^, while Aoyama et al. reported that SRS plus WBRT or SRS alone were associated with a lower recurrence rate, relative to WBRT alone^4^. Recent concerns regarding cognitive decline and decreased quality of life after WBRT have also seemed to support the use of SRS. We also observed that SRS was associated with a survival benefit relative to WBRT, suggesting that SRS may be the preferred RT modality for BM patients. We also cautiously suggest that active treatment is needed for BM patients, as patients who received WBRT plus SRS had better overall survival rates than those who received SRS alone or WBRT alone. Nevertheless, these results may also reflect the poorer performance status of BM patients who receive WBRT, relative to patients who receive SRS.

The major strengths of this study are the large Korean sample of BM patients with long-term follow-up data from the nationally representative NHIS database. However, the present study also has some limitations. First, the study involved a retrospective analysis of claims data, and overall survival was evaluated based on crude mortality rather than cancer-related mortality. Second, although the coding is reasonably accurate, the possibility of incomplete medical records suggests that the proportion of BM patients might have been underestimated. Third, the NHIS data do not include systematic information regarding RT-related adverse events, which could not be evaluated in the present study, although these factors can affect the long-term survival of cancer patients. Fourth, we could not pool data related to the best supportive care, hippocampal-sparing WBRT, or performance status based on the ECOG and KPS systems. Moreover, even with PSM corrections, the results were not robust because they were not properly distributed. Finally, the major limitation is the selection bias that can occur by excluding less than five fractions of RT. This raises questions about the reliability of these results as they create more favorable conclusions for the RT. Additionally, even if PSM were performed, there was still potential bias due to the lack of accurate comparative analysis of other prognostic variables, such as molecular markers, comorbidities, and status of cancer progression. Nevertheless, our study is the first investigation to demonstrate the effects of RT for brain metastases through a population-based cohort study.

In conclusion, this nationally representative cohort study revealed that Korean patients who received RT for BM had a lower crude mortality rate for 2.6 years after the RT, relative to patients who did not receive RT. In addition, RT was associated with a median survival difference of 3.7 months among patients with BM from lung cancer. Furthermore, it appears that SRS may be better than WBRT for treating patients with BM.

## Supplementary Information

Supplementary Information

## Data Availability

The datasets generated for and/or analyzed in the current study are available from the corresponding author upon reasonable request.
