# Supplementary material for: Radiotherapy for brain metastasis and long-term survival
Source: Sci Rep. 2021 Apr 13;11:8046. doi: 10.1038/s41598-021-87357-x (PMC8044241; doi:10.1038/s41598-021-87357-x)
Supplement: Supplementary file 1 — Supplementary Information [file 41598_2021_87357_MOESM1_ESM.pdf]

SUPPLEMENTAL MATERIAL

Scientific Reports

Radiotherapy for brain metastasis and long-term survival

Kawngwoo Park [1�], Gi Hwan Bae [2,7�], Woo Kyung Kim [1], Chan-Jong Yoo [1], Cheol Wan Park [3], Soo-Ki Kim [4], Jihye Cha [5], Jin Wook Kim [6], Jaehun Jung [2,7,*]

[1] Department of Neurosurgery Gachon University Gil Medical Center, Incheon, 21565, Republic of Korea

[2] Department of Preventive Medicine, Gachon University College of Medicine, Incheon, 21565, Republic of Korea

[3] Department of Emergency Medicine, Gachon University Gil Medical Center, Incheon, 21565, Republic of Korea

[4] Department of Microbiology, Wonju College of Medicine, Yonsei University, Wonju, 26426, Republic of Korea

[5] Department of Radiation Oncology, Wonju College of Medicine, Yonsei University, Wonju, 26426, Republic of Korea

[6] Department of Neurosurgery, Seoul National University College of Medicine, Seoul, 03080, Republic of Korea

[7] Gil Artificial Intelligence & Bigdata Convergence Center, Gachon University Gil Medical Center, Incheon, 21565 Republic of Korea

� Contributed equally and should be considered co-first authors.

* Correspondence: eastside1st@gmail.com; Tel.: +82-10-6359-3201

Supplemental Table 1. International Classification of Diseases 10th Revision (ICD-10) mapping for Charlson Comorbidity Index.[1]

Diseases	ICD-10 codes	Weight
Myocardial infarction	I21, I22, I252	1
Congestive heart failure	I43, I50, I099, I110, I130, I132, I255, I420, I425, I426, I427, I428, I429, P290	1
Peripheral vascular disease	I70, I71, I731, I738, I739, I771, I790, I792, K551, K558, K559, Z958, Z959	1
Cerebrovascular disease	G45, G46, I60 ~ I69, H340	1
Dementia	F00 ~ F03, G30, F051, G311	1
Chronic Obstructive Pulmonary Disease	J40 ~ J47, J60 ~ J67, I278, I279, J684, J701, J703	1
Connective Tissue Disease	M05, M32 ~ M34, M06, M315, M351, M353, M360	1
Peptic Ulcer Disease	K25 ~ K28	1
Mild Liver Disease	B18, K73, K74, K700, K701 ~ K703, K709, K717, K713, K714, K715, K760, K762 ~ K764, K768, K769, Z944	1
Diabetes without complications	E100, E101, E106, E108 ~ E111, E116, E118 ~ E121, E126, E128 ~ E131, E136, E138 ~ E141, E146, E148, E149	1
Diabetes with complications	E102 ~ E105, E107, E112 ~ E115, E117, E122 ~ E125, E127, E132 ~ E135, E137, E142 ~ E145, E147	2
Paraplegia and Hemiplegia	G81, G82, G041, G114, G801, G802, G830 ~ G834, G839	2
Renal Disease	N18, N19, N052 ~ N057, N250, I120, I131, N032 ~ N037, Z490, Z491, Z492, Z940, Z992	2
Cancer	C00 ~ C26, C30 ~ C34, C37 ~ C41, C43, C45 ~ C58, C60 ~ C76, C81 ~ C85, C88, C90 ~ C97	2
Moderate or Severe Liver Disease	K704, K711, K721, K729, K765, K766, K767, I850, I859, I864, I982	3
Metastatic Carcinoma	C77 ~ C80	6
Acquired immune deficiency syndrome (AIDS)	B20 ~ B22, B24	6

Note that all variables showed statistically significant differences at a p-value of 1%. In addition, all variables except sex differed in the probabilistic approach using a Bayesian factor.
SD, standard deviation; CCI, Charlson Comorbidity Index.

Supplemental Table 2. Chemotherapy codes registered with National healthcare insurance service system

Main code	Product code	ATC code	ATC code name
364100AGN	645400880	L01BC53	tegafur, combinations
388501ACH	652600810	L01XX	Other antineoplastic agents
388502ACH	652600820	L01XX	Other antineoplastic agents
412701ATB	645403950	L01XE01	imatinib
412701ATB	643506550	L01XE01	imatinib
412701ATB	647803790	L01XE01	imatinib
412701ATB	640006540	L01XE01	imatinib
412701ATB	644913540	L01XE01	imatinib
412701ATB	641906020	L01XE01	imatinib
412701ATB	642506940	L01XE01	imatinib
412701ATB	653600010	L01XE01	imatinib
412701ATB	643306580	L01XE01	imatinib
412701ATB	646802550	L01XE01	imatinib
412702ATB	641906040	L01XE01	imatinib
412702ATB	642506920	L01XE01	imatinib
412703ATB	645403960	L01XE01	imatinib
412703ATB	643506530	L01XE01	imatinib
412703ATB	640006530	L01XE01	imatinib
412703ATB	647803780	L01XE01	imatinib
412703ATB	642506930	L01XE01	imatinib
412703ATB	643306560	L01XE01	imatinib
412703ATB	641906030	L01XE01	imatinib
412703ATB	646802560	L01XE01	imatinib
412703ATB	644913550	L01XE01	imatinib
412704ATB	643506920	L01XE01	imatinib
452400ACH	649807210	L01BC53	tegafur, combinations
452400ACH	645401930	L01BC53	tegafur, combinations
452500ACH	649807220	L01BC53	tegafur, combinations
452500ACH	645401940	L01BC53	tegafur, combinations
453001ATB	648507340	L01XE02	gefitinib
453001ATB	641805910	L01XE02	gefitinib
453001ATB	642905710	L01XE02	gefitinib

453001ATB	643306980	L01XE02	gefitinib
453001ATB	643507210	L01XE02	gefitinib
453001ATB	650700460	L01XE02	gefitinib
477401ATB	626900760	L01XE03	erlotinib
477401ATB	640006640	L01XE03	erlotinib
477401ATB	642905700	L01XE03	erlotinib
477401ATB	641805870	L01XE03	erlotinib
477401ATB	643507230	L01XE03	erlotinib
477401ATB	643306650	L01XE03	erlotinib
477401ATB	641906190	L01XE03	erlotinib
477401ATB	645000480	L01XE03	erlotinib
477402ATB	626900770	L01XE03	erlotinib
477402ATB	640006630	L01XE03	erlotinib
477402ATB	641805880	L01XE03	erlotinib
477402ATB	642905290	L01XE03	erlotinib
477402ATB	643306660	L01XE03	erlotinib
477402ATB	644308530	L01XE03	erlotinib
477402ATB	645000490	L01XE03	erlotinib
477403ATB	626900750	L01XE03	erlotinib
477403ATB	645000500	L01XE03	erlotinib
485605ATB	653601520	L01XE10	everolimus
485606ATB	653601530	L01XE10	everolimus
485607ATB	653602230	L01XE10	everolimus
485701ACH	052900070	L04AX02	thalidomide
485701ACH	670701660	L04AX02	thalidomide
485701ACH	626100130	L04AX02	thalidomide
485701ACH	691800010	L04AX02	thalidomide
485701ACH	652603550	L04AX02	thalidomide
485702ACH	052900060	L04AX02	thalidomide
485702ACH	626100120	L04AX02	thalidomide
487701ACH	648900470	L01XE04	sunitinib
487702ACH	648900480	L01XE04	sunitinib
487703ACH	648900490	L01XE04	sunitinib
488001ATB	641104700	L01XE05	sorafenib
493301ATB	658700210	L01XE06	dasatinib

493302ATB	658700220	L01XE06	dasatinib
493303ATB	658700230	L01XE06	dasatinib
493304ATB	658700510	L01XE06	dasatinib
493305ATB	658700680	L01XE06	dasatinib
507501ATB	653602840	L01XE07	lapatinib
562601ACH	653601100	L01XE08	nilotinib
562602ACH	653602160	L01XE08	nilotinib
588201ACH	643307750	L04AX04	lenalidomide
588201ACH	641806390	L04AX04	lenalidomide
588201ACH	691800050	L04AX04	lenalidomide
588201ATB	622701000	L04AX04	lenalidomide
588202ACH	643307770	L04AX04	lenalidomide
588202ACH	641806410	L04AX04	lenalidomide
588202ACH	691800060	L04AX04	lenalidomide
588202ATB	622701010	L04AX04	lenalidomide
588203ACH	643307780	L04AX04	lenalidomide
588203ACH	641806400	L04AX04	lenalidomide
588203ACH	691800070	L04AX04	lenalidomide
588203ATB	622701030	L04AX04	lenalidomide
588204ACH	643307800	L04AX04	lenalidomide
588204ACH	641806380	L04AX04	lenalidomide
588204ACH	691800040	L04AX04	lenalidomide
588204ATB	622700980	L04AX04	lenalidomide
588205ACH	643307810	L04AX04	lenalidomide
588205ACH	641806360	L04AX04	lenalidomide
588205ACH	691800160	L04AX04	lenalidomide
588205ATB	622700970	L04AX04	lenalidomide
588206ACH	643307790	L04AX04	lenalidomide
588206ACH	641806370	L04AX04	lenalidomide
588206ACH	691800150	L04AX04	lenalidomide
588206ATB	622700990	L04AX04	lenalidomide
588207ACH	643307760	L04AX04	lenalidomide
588207ACH	641806420	L04AX04	lenalidomide
588207ACH	691800140	L04AX04	lenalidomide
588207ATB	622701020	L04AX04	lenalidomide

611801ATB	653602870	L01XE11	pazopanib
611802ATB	653602810	L01XE11	pazopanib
617501ACH	648902080	L01XE16	crizotinib
617502ACH	648902070	L01XE16	crizotinib
617701ACH	641704390	L01XE	Protein kinase inhibitors
617702ACH	641704380	L01XE	Protein kinase inhibitors
620402ATB	646902200	L02BX03	abiraterone
620501ATB	645001290	L01XE15	vemurafenib
621001ATB	648902130	L01XE17	axitinib
621002ATB	648902120	L01XE17	axitinib
623001ATB	653602400	L01XE18	ruxolitinib
623002ATB	653602390	L01XE18	ruxolitinib
623003ATB	653602380	L01XE18	ruxolitinib
624101ATB	693400200	L01XE12	vandetanib
624102ATB	693400210	L01XE12	vandetanib
624801ATB	641105790	L01XE21	regorafenib
626101ATB	653501430	L01XE13	afatinib
626102ATB	653501440	L01XE13	afatinib
626103ATB	653501450	L01XE13	afatinib
627401ACS	677200230	L02BB	Anti-androgens
628001ACH	691800130	L04AX06	pomalidomide
628002ACH	691800120	L04AX06	pomalidomide
628003ACH	691800110	L04AX06	pomalidomide
628004ACH	691800100	L04AX06	pomalidomide
628101ACH	646902020	L01XE27	ibrutinib
634401ACH	653602530	L01XE28	ceritinib
643501ACH	650700970	L01XX46	olaparib
645201ACH	621100150	L01XE29	lenvatinib
645202ACH	621100140	L01XE29	lenvatinib
645401ATB	653602690	L01XE25	trametinib
645402ATB	653602680	L01XE25	trametinib
652401ATB	643507570	L01XE40	olmutinib
652402ATB	643507580	L01XE40	olmutinib
652501ATB	650700990	L01XE35	osimertinib
652502ATB	650700980	L01XE35	osimertinib

655201ACH	648902930	L01XE33	palbociclib
655202ACH	648902940	L01XE33	palbociclib
655203ACH	648902920	L01XE33	palbociclib
656201ACH	645001430	L01XE36	alectinib
663101ACH	653602650	L01XE23	dabrafenib
663102ACH	653602660	L01XE23	dabrafenib
669601ATB	649900450	L01XE24	ponatinib
669602ATB	649900460	L01XE24	ponatinib
238430ASY	652604621	L03AX	Other immunostimulants
238431ASY	652604624	L03AX	Other immunostimulants
243001ACS	694800050	L01XX14	tretinoin
629001BIJ	052300041	M05BX04	denosumab
629002BIJ	052300061	M05BX04	denosumab
104501BIJ	653601261	L03AC01	aldesleukin
120630BIJ	649900341	L01AB01	busulfan
123730BIJ	641900161	L01XA02	carboplatin
123730BIJ	642501121	L01XA02	carboplatin
123730BIJ	648903234	L01XA02	carboplatin
123730BIJ	644303881	L01XA02	carboplatin
123731BIJ	641900151	L01XA02	carboplatin
123731BIJ	648903231	L01XA02	carboplatin
123731BIJ	642501131	L01XA02	carboplatin
123731BIJ	644303891	L01XA02	carboplatin
123732BIJ	648903232	L01XA02	carboplatin
123732BIJ	641900141	L01XA02	carboplatin
123732BIJ	642501141	L01XA02	carboplatin
123732BIJ	644303901	L01XA02	carboplatin
123733BIJ	641904421	L01XA02	carboplatin
123734BIJ	648903233	L01XA02	carboplatin
123735BIJ	641904431	L01XA02	carboplatin
134530BIJ	642502351	L01XA01	cisplatin
134530BIJ	644302861	L01XA01	cisplatin
134530BIJ	642905381	L01XA01	cisplatin
134533BIJ	642905391	L01XA01	cisplatin
134533BIJ	642502371	L01XA01	cisplatin

134534BIJ	644302871	L01XA01	cisplatin
134830BIJ	646900251	L01BB04	cladribine
139005BIJ	642201671	L01AA01	cyclophosphamide
139601BIJ	648900501	L01BC01	cytarabine
139602BIJ	648900511	L01BC01	cytarabine
139633BIJ	648903281	L01BC01	cytarabine
139637BIJ	648903291	L01BC01	cytarabine
139631BIJ	648903311	L01BC01	cytarabine
139632BIJ	644902935	L01BC01	cytarabine
139634BIJ	644913681	L01BC01	cytarabine
139635BIJ	644913691	L01BC01	cytarabine
139636BIJ	644913701	L01BC01	cytarabine
139638BIJ	644914101	L01BC01	cytarabine
139901BIJ	644300661	L01AX04	dacarbazine
139902BIJ	644300671	L01AX04	dacarbazine
148341BIJ	622700311	L01CD02	docetaxel
148342BIJ	622700321	L01CD02	docetaxel
148340BIJ	641905871	L01CD02	docetaxel
148344BIJ	641905841	L01CD02	docetaxel
148344BIJ	642506171	L01CD02	docetaxel
148344BIJ	643306431	L01CD02	docetaxel
148344BIJ	652000861	L01CD02	docetaxel
148344BIJ	649806881	L01CD02	docetaxel
148348BIJ	641905851	L01CD02	docetaxel
148348BIJ	642506181	L01CD02	docetaxel
148348BIJ	643306441	L01CD02	docetaxel
148348BIJ	652000871	L01CD02	docetaxel
148348BIJ	649806891	L01CD02	docetaxel
148349BIJ	642103821	L01CD02	docetaxel
148346BIJ	648506301	L01CD02	docetaxel
148346BIJ	642903781	L01CD02	docetaxel
148346BIJ	645403351	L01CD02	docetaxel
148346BIJ	643505511	L01CD02	docetaxel
148346BIJ	641804771	L01CD02	docetaxel
148350BIJ	642903791	L01CD02	docetaxel

148350BIJ	648506311	L01CD02	docetaxel
148350BIJ	643505521	L01CD02	docetaxel
148350BIJ	645403361	L01CD02	docetaxel
148350BIJ	641804781	L01CD02	docetaxel
148351BIJ	645403551	L01CD02	docetaxel
151901BIJ	658600771	L01BC	Pyrimidine analogues
157131BIJ	641903341	L01CB01	etoposide
157131BIJ	626900331	L01CB01	etoposide
157131BIJ	642505141	L01CB01	etoposide
157132BIJ	641903351	L01CB01	etoposide
160101BIJ	648903141	L01BB05	fludarabine
160101BIJ	659700941	L01BB05	fludarabine
160101BIJ	693400041	L01BB05	fludarabine
161430BIJ	644902301	L01BC02	fluorouracil
161431BIJ	644902311	L01BC02	fluorouracil
161432BIJ	644902321	L01BC02	fluorouracil
164930BIJ	658600031	L01BC05	gemcitabine
164930BIJ	670800261	L01BC05	gemcitabine
164930BIJ	648503051	L01BC05	gemcitabine
164930BIJ	642503081	L01BC05	gemcitabine
164930BIJ	642102171	L01BC05	gemcitabine
164930BIJ	647802091	L01BC05	gemcitabine
164930BIJ	643302541	L01BC05	gemcitabine
164930BIJ	643500101	L01BC05	gemcitabine
164930BIJ	643307191	L01BC05	gemcitabine
164930BIJ	648903302	L01BC05	gemcitabine
164931BIJ	658600041	L01BC05	gemcitabine
164931BIJ	648503041	L01BC05	gemcitabine
164931BIJ	670800271	L01BC05	gemcitabine
164931BIJ	642102161	L01BC05	gemcitabine
164931BIJ	642505041	L01BC05	gemcitabine
164931BIJ	647802081	L01BC05	gemcitabine
164931BIJ	643302551	L01BC05	gemcitabine
164931BIJ	643500091	L01BC05	gemcitabine
164931BIJ	643307181	L01BC05	gemcitabine

164931BIJ	648903301	L01BC05	gemcitabine
164932BIJ	644303581	L01BC05	gemcitabine
164932BIJ	648903303	L01BC05	gemcitabine
167201BIJ	650700491	L02AE03	goserelin
167202BIJ	650700481	L02AE03	goserelin
173301BIJ	642202521	L01AA06	ifosfamide
175530BIJ	645000151	L03AB04	interferon alfa-2a
175630BIJ	655501271	L03AB05	interferon alfa-2b
175631BIJ	655501261	L03AB05	interferon alfa-2b
177430BIJ	650901741	L01XX19	irinotecan
177430BIJ	641901621	L01XX19	irinotecan
177430BIJ	645402321	L01XX19	irinotecan
177430BIJ	641802561	L01XX19	irinotecan
177430BIJ	648503461	L01XX19	irinotecan
177430BIJ	642102211	L01XX19	irinotecan
177430BIJ	640003402	L01XX19	irinotecan
177430BIJ	643502131	L01XX19	irinotecan
177430BIJ	670500711	L01XX19	irinotecan
177430BIJ	648903261	L01XX19	irinotecan
177431BIJ	650901751	L01XX19	irinotecan
177431BIJ	641901631	L01XX19	irinotecan
177431BIJ	645402331	L01XX19	irinotecan
177431BIJ	648503471	L01XX19	irinotecan
177431BIJ	642102221	L01XX19	irinotecan
177431BIJ	641802571	L01XX19	irinotecan
177431BIJ	640003412	L01XX19	irinotecan
177431BIJ	643502141	L01XX19	irinotecan
177431BIJ	670500721	L01XX19	irinotecan
177431BIJ	648903271	L01XX19	irinotecan
177433BIJ	645402521	L01XX19	irinotecan
177433BIJ	648503481	L01XX19	irinotecan
177433BIJ	642102231	L01XX19	irinotecan
177433BIJ	641802591	L01XX19	irinotecan
177435BIJ	645402531	L01XX19	irinotecan
177435BIJ	648503491	L01XX19	irinotecan

177435BIJ	642102241	L01XX19	irinotecan
181401BIJ	666300241	L01XX02	asparaginase
182602BIJ	641601191	L02AE02	leuprorelin
182602BIJ	641601192	L02AE02	leuprorelin
182602BIJ	653400451	L02AE02	leuprorelin
182602BIJ	696300391	L02AE02	leuprorelin
182602BIJ	696300381	L02AE02	leuprorelin
182602BIJ	624900021	L02AE02	leuprorelin
182604BIJ	696300401	L02AE02	leuprorelin
182605BIJ	655601441	L02AE02	leuprorelin
182606BIJ	655601451	L02AE02	leuprorelin
182608BIJ	655601471	L02AE02	leuprorelin
182610BIJ	624900091	L02AE02	leuprorelin
182611BIJ	696300481	L02AE02	leuprorelin
182630BIJ	653400461	L02AE02	leuprorelin
189902BIJ	643903051	L01AA03	melphalan
192142BIJ	648902971	L01BA01	methotrexate
192143BIJ	648902991	L01BA01	methotrexate
192139BIJ	648903011	L01BA01	methotrexate
192139BIJ	642101491	L01BA01	methotrexate
192139BIJ	644305914	L01BA01	methotrexate
192139BIJ	644901641	L01BA01	methotrexate
192141BIJ	648903013	L01BA01	methotrexate
192141BIJ	644901631	L01BA01	methotrexate
192141BIJ	642101501	L01BA01	methotrexate
196530BIJ	658600411	L01DB07	mitoxantrone
198001BIJ	664000261	L01AC01	thiotepa
198003BIJ	664000271	L01AC01	thiotepa
205803BIJ	645402431	L01XA03	oxaliplatin
205803BIJ	641904461	L01XA03	oxaliplatin
205803BIJ	648504561	L01XA03	oxaliplatin
205830BIJ	693500311	L01XA03	oxaliplatin
205830BIJ	645402411	L01XA03	oxaliplatin
205830BIJ	641903081	L01XA03	oxaliplatin
205830BIJ	652000371	L01XA03	oxaliplatin

205830BIJ	677100161	L01XA03	oxaliplatin
205830BIJ	642102941	L01XA03	oxaliplatin
205830BIJ	641802441	L01XA03	oxaliplatin
205830BIJ	643307351	L01XA03	oxaliplatin
205830BIJ	658602481	L01XA03	oxaliplatin
205830BIJ	643300972	L01XA03	oxaliplatin
205830BIJ	643504131	L01XA03	oxaliplatin
205830BIJ	622700371	L01XA03	oxaliplatin
205830BIJ	648502811	L01XA03	oxaliplatin
205830BIJ	648903171	L01XA03	oxaliplatin
205832BIJ	648504571	L01XA03	oxaliplatin
205832BIJ	648903173	L01XA03	oxaliplatin
205834BIJ	693500321	L01XA03	oxaliplatin
205834BIJ	645402421	L01XA03	oxaliplatin
205834BIJ	641803771	L01XA03	oxaliplatin
205834BIJ	641903091	L01XA03	oxaliplatin
205834BIJ	648502821	L01XA03	oxaliplatin
205834BIJ	622700381	L01XA03	oxaliplatin
205834BIJ	652000381	L01XA03	oxaliplatin
205834BIJ	642102951	L01XA03	oxaliplatin
205834BIJ	658602491	L01XA03	oxaliplatin
205834BIJ	643307361	L01XA03	oxaliplatin
205834BIJ	643300942	L01XA03	oxaliplatin
205834BIJ	643504321	L01XA03	oxaliplatin
205834BIJ	648903172	L01XA03	oxaliplatin
207830BIJ	668100741	L01CD01	paclitaxel
207830BIJ	648902961	L01CD01	paclitaxel
207830BIJ	643503191	L01CD01	paclitaxel
207830BIJ	644900461	L01CD01	paclitaxel
207830BIJ	658700311	L01CD01	paclitaxel
207830BIJ	622700051	L01CD01	paclitaxel
207830BIJ	622700111	L01CD01	paclitaxel
207830BIJ	648503851	L01CD01	paclitaxel
207831BIJ	668100751	L01CD01	paclitaxel
207831BIJ	643503201	L01CD01	paclitaxel

207831BIJ	648503861	L01CD01	paclitaxel
207831BIJ	644302891	L01CD01	paclitaxel
207831BIJ	622700061	L01CD01	paclitaxel
207831BIJ	622700121	L01CD01	paclitaxel
207831BIJ	644900471	L01CD01	paclitaxel
207831BIJ	648902962	L01CD01	paclitaxel
207832BIJ	648902963	L01CD01	paclitaxel
207832BIJ	643503211	L01CD01	paclitaxel
207832BIJ	644302901	L01CD01	paclitaxel
207833BIJ	648503871	L01CD01	paclitaxel
207835BIJ	648902964	L01CD01	paclitaxel
207835BIJ	648503881	L01CD01	paclitaxel
211130BIJ	684200161	L03AX	Other immunostimulants
228130BIJ	659900551	L01XX	Other antineoplastic agents
241901BIJ	644308261	L01XX17	topotecan
241901BIJ	653602791	L01XX17	topotecan
242801BIJ	623800041	L01XC03	trastuzumab
242802BIJ	051500031	L01XC03	trastuzumab
242802BIJ	623800031	L01XC03	trastuzumab
242802BIJ	645000611	L01XC03	trastuzumab
242830BIJ	645001361	L01XC03	trastuzumab
244902BIJ	652500021	L02AE04	triptorelin
244902BIJ	681400031	L02AE04	triptorelin
244930BIJ	652500031	L02AE04	triptorelin
247830BIJ	626900041	L01CA01	vinblastine
247830BIJ	644301321	L01CA01	vinblastine
248030BIJ	648902981	L01CA02	vincristine
248030BIJ	658600481	L01CA02	vincristine
248031BIJ	648903111	L01CA02	vincristine
248230BIJ	642200051	L01CA04	vinorelbine
248230BIJ	648903241	L01CA04	vinorelbine
248231BIJ	642200071	L01CA04	vinorelbine
248231BIJ	648903251	L01CA04	vinorelbine
422603BIJ	645001381	L01XC02	rituximab
422632BIJ	645001421	L01XC02	rituximab

422630BIJ	623800061	L01XC02	rituximab
422630BIJ	645000221	L01XC02	rituximab
422631BIJ	623800051	L01XC02	rituximab
422631BIJ	645000231	L01XC02	rituximab
452801BIJ	643303471	L01XX	Other antineoplastic agents
463301BIJ	052500011	L01XX32	bortezomib
463301BIJ	643306901	L01XX32	bortezomib
463301BIJ	652606391	L01XX32	bortezomib
463301BIJ	641906311	L01XX32	bortezomib
463301BIJ	622700891	L01XX32	bortezomib
463301BIJ	646900441	L01XX32	bortezomib
463302BIJ	622700961	L01XX32	bortezomib
463302BIJ	641906331	L01XX32	bortezomib
463302BIJ	644309151	L01XX32	bortezomib
463303BIJ	652606381	L01XX32	bortezomib
467501BIJ	681400041	L02AE04	triptorelin
467502BIJ	681400141	L02AE04	triptorelin
481203BIJ	643306881	L01BA04	pemetrexed
481203BIJ	642506881	L01BA04	pemetrexed
481203BIJ	643507291	L01BA04	pemetrexed
481203BIJ	642905401	L01BA04	pemetrexed
481203BIJ	641805851	L01BA04	pemetrexed
481230BIJ	642904461	L01BA04	pemetrexed
481230BIJ	642507191	L01BA04	pemetrexed
481230BIJ	640006881	L01BA04	pemetrexed
481230BIJ	670800951	L01BA04	pemetrexed
481230BIJ	670500801	L01BA04	pemetrexed
481230BIJ	641805321	L01BA04	pemetrexed
481230BIJ	648903121	L01BA04	pemetrexed
481231BIJ	670500791	L01BA04	pemetrexed
481231BIJ	642904471	L01BA04	pemetrexed
481231BIJ	642506871	L01BA04	pemetrexed
481231BIJ	643307261	L01BA04	pemetrexed
481231BIJ	643306351	L01BA04	pemetrexed
481231BIJ	640006911	L01BA04	pemetrexed

481231BIJ	670800141	L01BA04	pemetrexed
481231BIJ	644913901	L01BA04	pemetrexed
481231BIJ	641805311	L01BA04	pemetrexed
481231BIJ	643507251	L01BA04	pemetrexed
481231BIJ	648903151	L01BA04	pemetrexed
481232BIJ	622700711	L01BA04	pemetrexed
481232BIJ	648903131	L01BA04	pemetrexed
481235BIJ	670500811	L01BA04	pemetrexed
481235BIJ	643307271	L01BA04	pemetrexed
481233BIJ	642507241	L01BA04	pemetrexed
481234BIJ	642507251	L01BA04	pemetrexed
481239BIJ	642507491	L01BA04	pemetrexed
484301BIJ	622701071	L01BC07	azacitidine
484301BIJ	641906761	L01BC07	azacitidine
484301BIJ	691800031	L01BC07	azacitidine
484302BIJ	622701041	L01BC07	azacitidine
495601BIJ	641906741	L01BC08	decitabine
495601BIJ	646901301	L01BC08	decitabine
495602BIJ	622701061	L01BC08	decitabine
503701BIJ	691800091	L01CD01	paclitaxel
554330BIJ	645000361	L01XC07	bevacizumab
554331BIJ	645000671	L01XC07	bevacizumab
556430BIJ	661700551	L01XC06	cetuximab
568230BIJ	648902371	L01XE09	temsirolimus
588430BIJ	052900081	L01XX27	arsenic trioxide
588430BIJ	679700231	L01XX27	arsenic trioxide
613901BIJ	652000901	L01CD04	cabazitaxel
614330BIJ	693400061	L01BB06	clofarabine
614601BIJ	621100011	L01AA09	bendamustine
614602BIJ	621100021	L01AA09	bendamustine
621330BIJ	621100031	L01XX41	eribulin
624401BIJ	652500341	L02BX02	degarelix
624402BIJ	652500352	L02BX02	degarelix
624501BIJ	696300191	L01XC12	brentuximab vedotin
624601BIJ	645001321	L01XC13	pertuzumab

626001BIJ	645001331	L01XC14	trastuzumab emtansine
626002BIJ	645001341	L01XC14	trastuzumab emtansine
628901BIJ	645001371	L01XC15	obinutuzumab
638401BIJ	050400011	L01XC17	nivolumab
638402BIJ	050400021	L01XC17	nivolumab
639001BIJ	655501901	L01XC18	pembrolizumab
639301BIJ	670801011	L01XC21	ramucirumab
639302BIJ	670801021	L01XC21	ramucirumab
647701BIJ	052300031	L01XC19	blinatumomab
647801BIJ	052300081	L01XX45	carfilzomib
647802BIJ	052300071	L01XX45	carfilzomib
647901BIJ	646902081	L04AC11	siltuximab
647902BIJ	646902091	L04AC11	siltuximab
657701BIJ	645001441	L01XC	Monoclonal antibodies
658801BIJ	652000931	L01XX44	aflibercept
658802BIJ	652000941	L01XX44	aflibercept
658901BIJ	670801091	L01XC27	olaratumab
114301BIJ	655501211	L03AX03	BCG vaccine

Supplemental Table 3. Cox regression of RT before/after PSM for patients with brain metastases (RT patients with more than 5 fractions RT)

	Non-RT		RT		Adjusted for age and sex			Adjusted for age, sex and CCI		
	All-cause mortality	Person-Years	All-cause mortality	Person-Years	HR	95%CI	p-value	HR	95%CI	p-value
	24483/28630 (85.50%)	44930.71	25503/28630 (89.10%)	44906.44	0.962	0.945-0.979	<.0001	0.961	0.945-0.978	<.0001
Sex										
Male	15393/17236 (89.31%)	21259.84	15202/16559 (91.81%)	21104.85	0.919	0.899-0.94	<.0001	0.919	0.898-0.94	<.0001
Female	9090/11394 (79.78%)	23670.87	10301/12071 (85.34%)	23801.59	1.033	1.004-1.062	0.0252	1.026	0.997-1.055	0.0767
Age										
20~29	79/122 (64.75%)	363.41	110/140 (78.57%)	315.28	1.279	0.956-1.712	0.0975	1.205	0.899-1.615	0.2131
30~39	598/835 (71.62%)	2174.5	860/1032 (83.33%)	2222.88	1.172	1.055-1.301	0.0031	1.122	1.01-1.246	0.0322
40~49	2373/3104 (76.45%)	7141.72	2927/3529 (82.94%)	7438.34	1.025	0.971-1.082	0.3686	1.001	0.948-1.057	0.9619
50~59	5798/7147 (81.12%)	12859.23	6551/7589 (86.32%)	13220.63	0.972	0.938-1.007	0.118	0.968	0.934-1.003	0.0723
60~69	8450/9650 (87.56%)	13737.78	8633/9461 (91.25%)	13616.16	0.942	0.914-0.971	<.0001	0.942	0.914-0.971	<.0001
70~79	7185/7772 (92.45%)	8654.08	6422/6879 (93.36%)	8093.15	0.917	0.887-0.949	<.0001	0.913	0.883-0.945	<.0001
Medical aid beneficiary	1563/1857 (84.17%)	2897.77	1630/1854 (87.92%)	2729.71	-	-	-	-	-	-
Medial facility										
Senior general hospital	9284/11133 (83.39%)	18758.78	10797/12173 (88.7%)	18643.92	1.01	0.982-1.039	0.4746	1.007	0.98-1.036	0.6104
Tertiary hospital	2294/2608 (87.96%)	4106.26	2267/2498 (90.75%)	3904.18	0.95	0.897-1.008	0.0881	0.951	0.897-1.009	0.0948
Secondary hospital	12904/14887 (86.68%)	22063.14	12434/13954 (89.11%)	22354.92	0.924	0.901-0.947	<.0001	0.924	0.902-0.947	<.0001
Local clinic	1/2 (50%)	2.53	5/5 (100%)	3.43	3.945	0.033-477.555	0.5749	-	-	-
Primary cancer										
Head and neck	518/675 (76.74%)	1589.21	585/682 (85.78%)	1582.39	1.121	0.996-1.262	0.0588	1.13	1.004-1.272	0.0434
Esophagus	388/432 (89.81%)	559.73	402/438 (91.78%)	448.38	1.054	0.916-1.213	0.4627	1.068	0.928-1.229	0.3615
Stomach	803/970 (82.78%)	1899.93	957/1064 (89.94%)	1525.3	1.113	1.012-1.224	0.0271	1.113	1.012-1.224	0.0271
Colorectal	2277/2875 (79.2%)	6198.84	2783/3108 (89.54%)	4715.75	1.291	1.221-1.365	<.0001	1.29	1.22-1.364	<.0001
Liver	1577/1783 (88.45%)	2159.44	1678/1805 (92.96%)	1746.16	1.095	1.021-1.173	0.0104	1.085	1.012-1.162	0.0216
Hepatobiliary	298/317 (94.01%)	330.2	289/306 (94.44%)	287.35	0.968	0.823-1.139	0.6961	0.96	0.816-1.129	0.6212
Pancreas	429/494 (86.84%)	770.09	464/534 (86.89%)	813.56	0.943	0.827-1.076	0.3857	0.945	0.828-1.078	0.3979

Pharynx		137/180 (76.11%)	521.08	165/194 (85.05%)	522	1.125	0.895-1.414	0.3124	1.137	0.904-1.43	0.2712
Lung		16719/18481 (90.47%)	21455.52	16284/17760 (91.69%)	24698.77	0.836	0.818-0.854	<.0001	0.836	0.818-0.854	<.0001
Breast		2328/3248 (71.67%)	9217.08	3019/3898 (77.45%)	9947.52	1.092	1.034-1.153	0.0015	1.061	1.005-1.121	0.0324
Cervix		213/298 (71.48%)	887.41	367/422 (86.97%)	756.98	1.491	1.257-1.767	<.0001	1.485	1.253-1.761	<.0001
Uterine		100/139 (71.94%)	386.19	153/186 (82.26%)	315.9	1.278	0.989-1.651	0.0607	1.281	0.991-1.655	0.0584
Ovary		306/394 (77.66%)	983.46	343/421 (81.47%)	889.27	1.08	0.926-1.261	0.3278	1.067	1.029-1.108	0.0005
Prostate		858/1036 (82.82%)	1893.63	846/951 (88.96%)	1476.72	1.156	1.051-1.272	0.0028	1.161	1.055-1.277	0.0023
Scrotum		20/33 (60.61%)	85.65	23/32 (71.88%)	68.21	1.229	0.672-2.25	0.5033	1.137	0.611-2.117	0.6858
Kidney		568/696 (81.61%)	1195.04	668/768 (86.98%)	1151.73	1.048	0.936-1.174	0.4122	1.047	0.935-1.173	0.4228
Bladder		248/310 (80%)	501.94	268/300 (89.33%)	395.94	1.161	0.976-1.381	0.092	1.141	0.958-1.357	0.1388
Thyroid		302/582 (51.89%)	2219.27	536/660 (81.21%)	1536.87	1.921	1.664-2.217	<.0001	1.929	1.67-2.227	<.0001
Operation		879/1091 (80.57%)	1839.1	1377/1641 (83.91%)	3576.72	0.838	0.77-0.913	<.0001	0.829	0.761-0.903	<.0001
Chemotherapy		12187/13731 (88.76%)	19494.12	13254/14798 (89.57%)	24270.85	0.879	0.857-0.901	<.0001	0.879	0.858-0.901	<.0001
Diagnostic year											
	2005	1554/1732 (89.72%)	3855.27	1423/1564 (90.98%)	3529.7	0.954	0.887-1.025	0.1976	0.954	0.887-1.025	0.1976
	2006	1701/2007 (84.75%)	5283.44	1728/1901 (90.9%)	4084.13	1.074	1.004-1.148	0.0379	1.073	1.004-1.148	0.0387
	2007	1736/2146 (80.89%)	5925.68	1808/2043 (88.5%)	4738.1	1.11	1.039-1.186	0.002	1.106	1.035-1.181	0.0029
	2008	1944/2197 (88.48%)	4154.91	1871/2045 (91.49%)	3856.64	0.951	0.893-1.014	0.1251	0.952	0.893-1.014	0.1276
	2009	2066/2323 (88.94%)	3826.37	2074/2289 (90.61%)	4326.28	0.888	0.835-0.944	0.0001	0.89	0.837-0.946	0.0002
	2010	2231/2532 (88.11%)	3974.66	2220/2436 (91.13%)	4141.45	0.902	0.851-0.957	0.0006	0.903	0.851-0.958	0.0007
	2011	2173/2453 (88.59%)	3549.94	2339/2587 (90.41%)	4260.85	0.878	0.827-0.931	<.0001	0.878	0.828-0.931	<.0001
	2012	2167/2474 (87.59%)	3252.36	2264/2461 (92.00%)	3345.68	0.938	0.884-0.995	0.0332	0.938	0.884-0.995	0.0333
	2013	2260/2656 (85.09%)	3288.67	2379/2616 (90.94%)	3283.75	1.007	0.951-1.067	0.8124	1.007	0.95-1.067	0.8188
	2014	2183/2573 (84.84%)	2890.7	2536/2871 (88.33%)	3511.67	0.961	0.907-1.018	0.1717	0.96	0.907-1.017	0.1652
	2015	2235/2683 (83.3%)	2584.4	2407/2810 (85.66%)	3097.91	0.91	0.859-0.964	0.0014	0.91	0.859-0.964	0.0014
	2016	2233/2854 (78.24%)	2344.3	2454/3007 (81.61%)	2730.27	0.953	0.9-1.009	0.1005	0.954	0.9-1.01	0.1052

Abbreviations : Non-RT: Non-radio therapy, RT: radio therapy, PSM: propensity score matching, CCI: Charlson Comorbidity Index, HR: Hazard ratio,

Supplemental Table 4. Cox regression of RT before/after PSM for patients with brain metastases (RT patients with at least one fraction RT)

	Non-RT		RT (≥1)		Adjusted for age, sex and psm p-value <0.05 variable		
	All-cause mortality	Person-Years	All-cause mortality	Person-Years	HR	95%CI	p-value
	20664/26175 (78.95%)	48413.51	23636/26175 (90.3%)	27908.6	1.335	1.31-1.36	<.0001
Sex							
Male	13670/16229 (84.23%)	23744.87	14973/16229 (92.26%)	14603.99	1.231	1.2-1.26	<.0001
Female	6994/9946 (70.32%)	24668.64	8663/9946 (87.1%)	13304.62	1.541	1.49-1.59	<.0001
Age							
20~29	53/94 (56.38%)	339.09	77/94 (81.91%)	175.87	2.153	1.46-3.18	0.0001
30~39	350/611 (57.28%)	2113.27	506/611 (82.82%)	1023.12	1.91	1.66-2.2	<.0001
40~49	1408/2223 (63.34%)	6875.23	1914/2223 (86.1%)	3281.69	1.825	1.7-1.96	<.0001
50~59	3589/5164 (69.5%)	12456.42	4465/5164 (86.46%)	6371.06	1.564	1.5-1.64	<.0001
60~69	6570/8268 (79.46%)	14912	7532/8268 (91.1%)	8771.89	1.32	1.28-1.37	<.0001
70~79	8694/9815 (88.58%)	11717.5	9142/9815 (93.14%)	8284.97	1.145	1.11-1.18	<.0001
Medical aid beneficiary	1835/2278 (80.55%)	3781.87	1854/2096 (88.45%)	2121.83	1.252	1.17-1.34	<.0001
Medial facility							
Senior general hospital	6424/8676 (74.04%)	19188.9	7808/8793 (88.8%)	9868.65	1.42	1.37-1.47	<.0001
Tertiary hospital	2252/2719 (82.82%)	4710.36	2251/2441 (92.22%)	2593.05	1.229	1.16-1.31	<.0001
Secondary hospital	11984/14771 (81.13%)	24484.13	13570/14933 (90.87%)	15434.66	1.301	1.27-1.33	<.0001
Local clinic	4/9 (44.44%)	30.11	7/8 (87.5%)	12.24	-	-	-
Primary cancer							
Head and neck	340/501 (67.86%)	1301.78	386/477 (80.92%)	1240.77	1.239	1.06-1.44	0.0061

	Non-RT		RT (1 – 4 fractions)		Adjusted for age, sex and psm p-value <0.05 variable		
	All-cause mortality	Person-Years	All-cause mortality	Person-Years	HR	95%CI	p-value
	18012/22907 (78.63%)	42842.02	20763/22907 (90.64%)	23399.11	1.399	1.37-1.43	<.0001
Sex							
Male	11941/14199 (84.1%)	20853.92	13219/14199 (93.1%)	11754.55	1.315	1.28-1.35	<.0001
Female	6071/8708 (69.72%)	21988.1	7544/8708 (86.63%)	11644.56	1.556	1.5-1.61	<.0001
Age							
20~29	41/70 (58.57%)	218.52	60/70 (85.71%)	88.39	1.785	1.1-2.9	0.0191
30~39	307/522 (58.81%)	1722.38	440/522 (84.29%)	767.76	1.866	1.6-2.17	<.0001
40~49	1303/2026 (64.31%)	6001.44	1738/2026 (85.78%)	2985.21	1.71	1.59-1.84	<.0001
50~59	3337/4790 (69.67%)	11413.99	4160/4790 (86.85%)	5634.82	1.582	1.51-1.66	<.0001
60~69	6078/7619 (79.77%)	13608.15	6970/7619 (91.48%)	7500.58	1.372	1.32-1.42	<.0001
70~79	6946/7880 (88.15%)	9877.55	7395/7880 (93.85%)	6422.34	1.247	1.21-1.29	<.0001
Medical aid beneficiary	1598/1985 (80.5%)	3400.63	1578/1760 (89.66%)	1720	1.37	1.28-1.47	<.0001
Medial facility							
Senior general hospital	5827/7903 (73.73%)	17497.29	7234/8163 (88.62%)	9049.45	1.409	1.36-1.46	<.0001
Tertiary hospital	2039/2460 (82.89%)	4231.26	2146/2326 (92.26%)	2382.87	1.252	1.18-1.33	<.0001
Secondary hospital	10145/12543 (80.88%)	21106.72	11379/12414 (91.66%)	11965.97	1.418	1.38-1.46	<.0001
Local clinic	1/1 (100%)	6.76	4/4 (100%)	0.82	-	-	-
Primary cancer							
Head and neck	309/446 (69.28%)	1123.54	338/405 (83.46%)	905.23	1.284	1.09-1.51	0.0024

	Non-RT		RT (≥5 fractions)		Adjusted for age, sex and psm p-value <0.05 variable		
	All-cause mortality	Person-Years	All-cause mortality	Person-Years	HR	95%CI	p-value
	12488/16182 (77.17%)	30352.61	14222/16182 (87.89%)	21566.21	1.457	1.37-1.48	<.0001
Sex							
Male	8195/9847 (83.22%)	14022.88	8858/9847 (89.96%)	11268.01	1.095	1.06-1.13	<.0001
Female	4293/6335 (67.77%)	16329.73	5364/6335 (84.67%)	10298.2	1.434	1.38-1.49	<.0001
Age							
20~29	23/37 (62.16%)	86.31	33/37 (89.19%)	43.18	1.443	0.74-2.82	0.2823
30~39	245/428 (57.24%)	1413.68	351/428 (82.01%)	790.25	1.692	1.43-2	<.0001
40~49	1083/1682 (64.39%)	4785.57	1394/1682 (82.88%)	3000.63	1.462	1.35-1.59	<.0001
50~59	2862/4066 (70.39%)	8981.13	3466/4066 (85.24%)	5904.56	1.336	1.27-1.41	<.0001
60~69	4512/5688 (79.32%)	9714.84	5077/5688 (89.26%)	7322.5	1.16	1.11-1.21	<.0001
70~79	3763/4281 (87.9%)	5371.09	3901/4281 (91.12%)	4505.1	1.052	1.01-1.1	0.0289
Medical aid beneficiary	988/1281 (77.13%)	2317.2	963/1113 (86.52%)	1384.11	1.193	1.09-1.31	0.0001
Medial facility							
Senior general hospital	4413/5940 (74.29%)	12268.44	5313/6014 (88.34%)	7337.08	1.332	1.28-1.39	<.0001
Tertiary hospital	1181/1458 (81%)	2661.04	1075/1232 (87.26%)	1881.72	1.065	0.98-1.16	0.1457
Secondary hospital	6890/8774 (78.53%)	15396.4	7829/8930 (87.67%)	12335.48	1.143	1.11-1.18	<.0001
Local clinic	4/10 (40%)	26.74	5/6 (83.33%)	11.92	-	-	-
Primary cancer							
Head and neck	268/400 (67%)	1042.39	297/371 (80.05%)	905.37	1.224	1.03-1.46	0.0215

Esophagus	251/301 (83.39%)	502.29	268/290 (92.41%)	294.28	1.334	1.11-1.6	0.002	226/273 (82.78%)	452.6	240/256 (93.75%)	219.38	1.473	1.22-1.78	<.0001	181/223 (81.17%)	397.38	244/273 (89.38%)	317.21	1.266	1.03-1.55	0.024
Stomach	1337/1926 (69.42%)	6396.93	1454/1579 (92.08%)	1691.48	1.83	1.69-1.98	<.0001	1174/1684 (69.71%)	5586.01	1192/1262 (94.45%)	1123.24	1.941	1.78-2.12	<.0001	594/780 (76.15%)	1882.8	502/577 (87%)	867.94	1.25	1.1-1.42	0.0006
Colorectal	1577/2317 (68.06%)	6640.25	2063/2316 (89.08%)	3039.6	1.769	1.65-1.89	<.0001	1402/2050 (68.39%)	5820.89	1832/2048 (89.45%)	2507.48	1.826	1.7-1.96	<.0001	1064/1590 (66.92%)	4500.96	1323/1513 (87.44%)	2263.01	1.623	1.49-1.76	<.0001
Liver	1449/1718 (84.34%)	2534.85	1413/1508 (93.7%)	1134.74	1.438	1.33-1.55	<.0001	1267/1502 (84.35%)	2184.19	1245/1314 (94.75%)	869.91	1.538	1.42-1.67	<.0001	954/1147 (83.17%)	1675.3	861/935 (92.09%)	888.73	1.233	1.12-1.35	<.0001
Hepatobiliary	347/403 (86.1%)	550.53	380/399 (95.24%)	288.06	1.257	1.08-1.47	0.0036	295/336 (87.8%)	457.44	273/282 (96.81%)	180.02	1.399	1.17-1.67	0.0002	195/220 (88.64%)	269.6	177/193 (91.71%)	192.22	0.927	0.75-1.15	0.4982
Pancreas	594/708 (83.9%)	1187.5	523/593 (88.2%)	703.84	1.02	0.9-1.15	0.745	483/572 (84.44%)	930.29	438/489 (89.57%)	507.55	1.148	1-1.31	0.0452	289/358 (80.73%)	636.97	248/295 (84.07%)	446.45	0.938	0.79-1.12	0.4747
Pharynx	83/123 (67.48%)	408.28	109/136 (80.15%)	377.46	0.976	0.72-1.32	0.8761	79/114 (69.3%)	361.99	90/110 (81.82%)	306.04	1.007	0.73-1.39	0.9666	63/97 (64.95%)	344.37	91/112 (81.25%)	309.81	1.252	0.87-1.8	0.2244
Lung	10976/12976 (84.59%)	16617.32	12764/14056 (90.81%)	14914.3	1.07	1.04-1.1	<.0001	9910/11782 (84.11%)	15453.05	11879/13053 (91.01%)	13180.9	1.149	1.12-1.18	<.0001	7019/8522 (82.36%)	11677.71	8251/9232 (89.37%)	11662.81	1.022	0.99-1.06	0.1762
Breast	950/1767 (53.76%)	6919.32	1163/1582 (73.51%)	3796.85	1.629	1.49-1.78	<.0001	902/1696 (53.18%)	6745.31	1210/1569 (77.12%)	3420.93	1.778	1.63-1.94	<.0001	864/1625 (53.17%)	6414.35	1179/1641 (71.85%)	4266.31	1.619	1.48-1.77	<.0001
Cervix	103/170 (60.59%)	647.15	143/169 (84.62%)	298.5	1.749	1.33-2.3	<.0001	89/150 (59.33%)	569.84	126/153 (82.35%)	264.19	1.947	1.45-2.61	<.0001	89/154 (57.79%)	609.95	147/183 (80.33%)	357.03	1.848	1.38-2.47	<.0001
Uterine	48/82 (58.54%)	298.02	72/83 (86.75%)	120.97	2.133	1.4-3.25	0.0004	47/78 (60.26%)	280.91	70/84 (83.33%)	134.02	2.185	1.42-3.37	0.0004	44/74 (59.46%)	269.88	58/77 (75.32%)	137.79	1.61	0.99-2.62	0.0552
Ovary	248/387 (64.08%)	1130.71	308/374 (82.35%)	659.21	1.649	1.38-1.97	<.0001	235/358 (65.64%)	1016.73	276/333 (82.88%)	567.17	1.592	1.33-1.91	<.0001	155/232 (66.81%)	625.88	141/183 (77.05%)	355.39	1.54	1.2-1.97	0.0006
Prostate	680/918 (74.07%)	2046.21	821/945 (86.88%)	1413.92	1.418	1.28-1.57	<.0001	573/778 (73.65%)	1739.04	680/765 (88.89%)	1014.83	1.565	1.39-1.76	<.0001	420/564 (74.47%)	1187.29	538/635 (84.72%)	1050.46	1.204	1.06-1.37	0.0057
Scrotum	11/29 (37.93%)	138.18	20/30 (66.67%)	64.92	4.54	1.35-15.26	0.0145	13/30 (43.33%)	148.7	16/22 (72.73%)	45.3	4.043	1.11-14.7	0.0339	9/17 (52.94%)	59.82	12/14 (85.71%)	22.58	1.687	0.22-13.03	0.6163
Kidney	399/561 (71.12%)	1349.96	416/482 (86.31%)	563.1	1.613	1.4-1.86	<.0001	373/528 (70.64%)	1298.28	428/491 (87.17%)	551.97	1.64	1.41-1.9	<.0001	218/298 (73.15%)	585.59	214/240 (89.17%)	283.49	1.524	1.25-1.86	<.0001
Bladder	229/315 (72.7%)	703.27	286/321 (89.1%)	354.61	1.526	1.27-1.83	<.0001	205/280 (73.21%)	623.45	233/257 (90.66%)	244.24	1.627	1.33-1.99	<.0001	133/183 (72.68%)	395.22	148/176 (84.09%)	241.56	1.203	0.93-1.56	0.1617
Thyroid	208/592 (35.14%)	2861.08	451/579 (77.89%)	1280.78	2.733	2.3-3.25	<.0001	180/484 (37.19%)	2249.78	337/444 (75.9%)	988.89	2.416	2-2.92	<.0001	131/369 (35.5%)	1694.3	252/321 (78.5%)	701.81	3.046	2.42-3.84	<.0001
Operation	509/712 (71.49%)	1205.38	541/697 (77.62%)	1361.67	0.928	0.82-1.05	0.2384	486/684 (71.05%)	1167.3	529/680 (77.79%)	1274.25	0.931	0.82-1.06	0.2791	393/559 (70.3%)	920.62	467/568 (82.22%)	946.03	1.021	0.89-1.17	0.7634
Chemotherapy	6999/8689 (80.55%)	12684.22	7715/8838 (87.29%)	10525.82	1.179	1.14-1.22	<.0001	6570/8200 (80.12%)	12174.64	7342/8450 (86.89%)	9997.84	1.196	1.16-1.24	<.0001	5487/6938 (79.09%)	10580.64	6582/7518 (87.55%)	9924.26	1.151	1.11-1.19	<.0001
Diagnostic year																					
2005	1793/2087 (85.91%)	5083.12	1687/1783 (94.62%)	2363.36	1.32	1.23-1.41	<.0001	1339/1580 (84.75%)	4216.64	1493/1566 (95.34%)	1855.7	1.526	1.41-1.65	<.0001	883/1057 (83.54%)	2993.15	885/957 (92.48%)	1717.53	1.253	1.14-1.38	<.0001
2006	1915/2509 (76.33%)	8464.6	2175/2326 (93.51%)	3182.52	1.535	1.44-1.64	<.0001	1828/2341 (78.09%)	7401.23	1838/1951 (94.21%)	2523.39	1.484	1.39-1.59	<.0001	994/1327 (74.91%)	4723.55	1026/1132 (90.64%)	2113.63	1.367	1.25-1.5	<.0001
2007	1781/2438 (73.05%)	8340.51	2105/2293 (91.8%)	3454.62	1.57	1.47-1.68	<.0001	1634/2198 (74.34%)	7198.39	1915/2040 (93.87%)	2653.81	1.57	1.47-1.68	<.0001	880/1236 (71.2%)	4413.37	963/1112 (86.6%)	2457.5	1.351	1.23-1.48	<.0001
2008	1881/2250 (83.6%)	4674.4	1949/2074 (93.97%)	2439.16	1.305	1.22-1.39	<.0001	1647/1994 (82.6%)	4386.22	1810/1906 (94.96%)	2018.55	1.447	1.35-1.55	<.0001	1030/1245 (82.73%)	2802.68	1005/1107 (90.79%)	1953.85	1.066	0.98-1.17	0.1573
2009	1791/2123 (84.36%)	3880.06	1931/2058 (93.83%)	2436.24	1.221	1.14-1.3	<.0001	1583/1885 (83.98%)	3543.15	1806/1912 (94.46%)	2071.62	1.305	1.22-1.4	<.0001	931/1119 (83.2%)	2312.64	983/1088 (90.35%)	1835.09	1.084	0.99-1.19	0.0825

2010	1827/2192 (83.35%)	3711.05	2023/2191 (92.33%)	2576.7	1.206	1.13-1.29	<.0001	1648/1965 (83.87%)	3290.45	1924/2049 (93.9%)	2125.46	1.302	1.22-1.39	<.0001	1105/1341 (82.4%)	2379.87	1167/1275 (91.53%)	1965.45	1.065	0.98-1.16	0.139
2011	1596/1910 (83.56%)	3027.46	1919/2069 (92.75%)	2257.95	1.247	1.17-1.33	<.0001	1434/1714 (83.66%)	2697.33	1724/1844 (93.49%)	1876.93	1.288	1.2-1.38	<.0001	1125/1341 (83.89%)	2144.41	1190/1305 (91.19%)	1852.93	1.044	0.96-1.13	0.3053
2012	1626/1959 (83%)	2659.7	1866/2011 (92.79%)	1894.26	1.297	1.21-1.39	<.0001	1358/1653 (82.15%)	2367.28	1606/1731 (92.78%)	1703.51	1.356	1.26-1.46	<.0001	1191/1456 (81.8%)	2120.42	1402/1528 (91.75%)	1706.63	1.157	1.07-1.25	0.0003
2013	1686/2106 (80.06%)	2660.06	2005/2190 (91.55%)	2024.14	1.308	1.22-1.4	<.0001	1462/1839 (79.5%)	2392.01	1728/1874 (92.21%)	1786.09	1.348	1.26-1.45	<.0001	1207/1532 (78.79%)	2074.89	1511/1666 (90.7%)	1792.42	1.257	1.16-1.36	<.0001
2014	1593/2018 (78.94%)	2232.1	2007/2232 (89.92%)	1939.7	1.282	1.2-1.37	<.0001	1420/1799 (78.93%)	2009.02	1681/1884 (89.23%)	1705.55	1.293	1.2-1.39	<.0001	1065/1399 (76.13%)	1681.13	1469/1679 (87.49%)	1659.96	1.234	1.14-1.34	<.0001
2015	1556/2098 (74.17%)	1928.28	2007/2329 (86.17%)	1715.24	1.295	1.21-1.38	<.0001	1296/1798 (72.08%)	1777.3	1682/1974 (85.21%)	1589.34	1.369	1.27-1.47	<.0001	1105/1531 (72.18%)	1491.92	1410/1677 (84.08%)	1405.68	1.244	1.15-1.35	<.0001
2016	1619/2485 (65.15%)	1752.17	1962/2619 (74.91%)	1624.7	1.304	1.22-1.39	<.0001	1363/2141 (63.66%)	1563.01	1556/2176 (71.51%)	1489.16	1.249	1.16-1.35	<.0001	972/1598 (60.83%)	1214.59	1211/1656 (73.13%)	1105.54	1.33	1.22-1.45	<.0001

Abbreviations : Non-RT: Non-radio therapy, RT: radio therapy, PSM: propensity score matching, HR: Hazard ratio,

Supplemental Table 5. Characteristics of study subjects according to the RT modality (RT patients with more than 5 fractions RT)

	Before PSM				After PSM								
	Radiosurgery	WBRT	Radiosurgery + WBRT	P-value	Radiosurgery	WBRT	P-value	Radiosurgery	Radiosurgery + WBRT	P-value	WBRT	Radiosurgery + WBRT	P-value
	7847	23899	5300		7642	7642		4823	4823		5245	5245	
Sex				<.0001			0.6815			0.0007			0.3386
Male	4622	12824	2650		4484	4459		2784	2619		2576	2625	
Female	3225	11075	2650		3158	3183		2039	2204		2669	2620	
Age	61.34±10.92	59.28±11.15	56.75±10.79	<.0001	61.33±10.89	61.30±10.74	0.8393	58.80±10.78	57.52±10.57	0.0006	57.24±11.19	56.80±10.74	0.0023
20~29	30	137	31		27	21		24	22		52	29	
30~39	254	1086	320		245	254		210	240		324	310	
40~49	906	3406	958		888	850		730	806		955	950	
50~59	2000	7017	1771		1950	1985		1505	1595		1616	1755	
60~69	2581	7412	1551		2520	2514		1560	1499		1544	1539	
70~79	2076	4841	669		2012	2018		794	661		754	662	
Medical aid beneficiary	363	1544	220	<.0001	354	329	0.3277	198	208	0.6121	376	219	<.0001
Medial facility				<.0001			0.05			0.005			0.0008
Senior general hospital	4228	10241	2874		4110	4258		2675	2644		2723	2841	
Tertiary hospital	903	1690	423		864	812		474	396		516	412	
Secondary hospital	2716	11963	2003		2668	2572		1674	1783		2006	1992	
Local clinic	0	5	0		0	0							
Primary cancer													
Head and neck	116	542	97	<.0001	116	93	0.1092	93	93	1	170	97	<.0001
Esophagus	55	404	58	<.0001	55	49	0.5549	51	55	0.696	83	58	0.034
Stomach	276	703	104	<.0001	272	244	0.2098	132	103	0.0555	184	104	<.0001
Colorectal	801	2144	618	<.0001	795	751	0.2379	585	575	0.7543	777	617	<.0001
Liver	427	1311	248	0.0582	423	395	0.3143	283	238	0.0427	361	248	<.0001
Hepatobiliary	61	232	21	0.0001	61	49	0.2508	32	21	0.1297	43	21	0.0058
Pancreas	121	384	64	0.1016	119	92	0.0612	72	61	0.3368	107	64	0.0009

Pharynx		29	182	21	<.0001	29	33	0.6107	19	20	0.8725	28	21	0.3162
Lung		5371	12599	3227	<.0001	5341	5525	0.001	3220	3182	0.4128	2784	3222	<.0001
Breast		605	4672	1186	<.0001	605	642	0.2743	591	779	<.0001	1200	1186	0.7444
Cervix		51	491	73	<.0001	51	42	0.3492	46	60	0.1715	126	73	0.0001
Uterine		29	167	39	0.0038	29	28	0.8944	27	30	0.6902	61	39	0.0271
Ovary		140	254	67	<.0001	138	116	0.1639	68	62	0.5962	98	67	0.015
Prostate		155	742	114	<.0001	154	131	0.169	113	112	0.9462	176	114	0.0002
Scrotum		10	19	6	0.4343	10	9	0.8184	8	6	0.5927	8	6	0.5927
Kidney		439	299	148	<.0001	394	273	<.0001	216	148	0.0003	169	141	0.1065
Bladder		82	212	28	0.0066	78	74	0.7444	36	27	0.2553	45	28	0.0459
Thyroid		170	456	126	0.0568	168	155	0.4647	108	111	0.8375	183	126	0.001
Operation		756	1072	743	<.0001	715	647	0.0535	601	638	0.2602	599	736	<.0001
Chemotherapy		4388	13296	3639	<.0001	4293	4382	0.1462	3070	3207	0.0034	3219	3604	<.0001
Diagnostic year					<.0001			0.998			0.1683			<.0001
	2005	299	1366	139		288	285		159	131		156	135	
	2006	416	1586	189		406	433		203	184		279	185	
	2007	474	1603	220		462	455		245	215		241	216	
	2008	604	1592	280		591	593		304	275		396	276	
	2009	695	1668	391		677	677		394	378		404	385	
	2010	755	1911	422		721	696		403	406		426	419	
	2011	754	2031	495		730	714		472	458		599	493	
	2012	606	2392	554		596	588		478	476		473	550	
	2013	715	2524	620		688	713		477	542		600	614	
	2014	773	2574	658		759	768		516	578		498	655	
	2015	803	2485	722		789	784		610	610		629	714	
	2016	953	2167	610		935	936		562	570		544	603	
CCI		5.21±2.06	4.78±2.15	2.09±1.91	<.0001	5.2±2.05	5.23±2.13	0.4294	5.16±2.09	5.11±1.94	0.1874	5.12±2.29	5.09±1.91	0.3872

Abbreviations : RT: radio therapy, PSM: propensity score matching, CCI: Charlson Comorbidity Index, WBRT, whole-brain radiotherapy

Note that all variables showed statistically significant differences at a p-value of 1%. In addition, all variables except sex differed in the probabilistic approach using a Bayesian factor.

Supplemental Table 6. Before PSM, characteristics of study subjects according to the RT modality (RT patients with at least one fraction RT)

	Radiosurgery		WBRT		Radiosurgery + WBRT		P-value
	N	%	N	%	N	%	
	5276		42187		7871		
Sex							
Male	3157	59.84	24003	56.9	4115	52.28	<.0001
Female	2119	40.16	18184	43.1	3756	47.72	
Age	62.61±10.76		60.49±11.11		57.40±10.82		<.0001
20~29	18	0.34	218	0.52	43	0.55	<.0001
30~39	142	2.69	1585	3.76	432	5.49	
40~49	504	9.55	5315	12.6	1360	17.28	
50~59	1234	23.39	11411	27.05	2537	32.23	
60~69	1773	33.61	13576	32.18	2359	29.97	
70~79	1605	30.42	10082	23.9	1140	14.48	
Medical aid beneficiary	231	4.38	2941	6.97	352	4.47	<.0001
Medial facility							
Senior general hospital	2941	55.74	16553	39.24	4112	52.24	<.0001
Tertiary hospital	624	11.83	3425	8.12	698	8.87	
Secondary hospital	1711	32.43	22205	52.63	3061	38.89	
Local clinic			4	0.01			
Primary cancer							
Head and neck	71	1.35	856	2.03	142	1.8	0.0021
Esophagus	40	0.76	605	1.43	73	0.93	<.0001
Stomach	205	3.89	1586	3.76	175	2.22	<.0001
Colorectal	572	10.84	3436	8.14	847	10.76	<.0001
Liver	277	5.25	2235	5.3	398	5.06	0.6784
Hepatobiliary	46	0.87	429	1.02	36	0.46	<.0001
Pancreas	90	1.71	729	1.73	95	1.21	0.0037
Pharynx	17	0.32	259	0.61	33	0.42	0.0056
Lung	3636	68.92	23309	55.25	4962	63.04	<.0001

Breast	303	5.74	6250	14.81	1488	18.9	<.0001
Cervix	28	0.53	627	1.49	96	1.22	<.0001
Uterine	18	0.34	238	0.56	50	0.64	0.0683
Ovary	98	1.86	463	1.1	109	1.38	<.0001
Prostate	112	2.12	1285	3.05	157	1.99	<.0001
Scrotum	7	0.13	38	0.09	9	0.11	0.5663
Kidney	315	5.97	629	1.49	272	3.46	<.0001
Bladder	58	1.1	388	0.92	52	0.66	0.0225
Thyroid	115	2.18	675	1.6	181	2.3	<.0001
Operation	473	8.97	1555	3.69	1026	13.04	<.0001
Chemotherapy	2845	53.92	21305	50.5	5182	65.84	<.0001
Diagnostic year							
2005	177	3.35	2887	6.84	261	3.32	<.0001
2006	244	4.62	3287	7.79	361	4.59	
2007	263	4.98	3371	7.99	431	5.48	
2008	349	6.61	3367	7.98	535	6.8	
2009	416	7.88	3408	8.08	670	8.51	
2010	447	8.47	3689	8.74	730	9.27	
2011	470	8.91	3600	8.53	779	9.9	
2012	415	7.87	3689	8.74	745	9.47	
2013	510	9.67	3800	9.01	825	10.48	
2014	575	10.9	3878	9.19	856	10.88	
2015	625	11.85	3765	8.92	900	11.43	
2016	785	14.88	3446	8.17	778	9.88	
CCI	5.11±2.19		4.31±2.50		5.08±2.0		<.0001

Abbreviations : RT: radio therapy, PSM: propensity score matching, CCI: Charlson Comorbidity Index, WBRT, whole-brain radiotherapy

Supplemental Table 7. After PSM, characteristics of study subjects according to the RT modality (RT patients with at least one fraction RT)

	Radiosurgery		WBRT		P-value	Radiosurgery		Radiosurgery + WBRT		P-value	WBRT		Radiosurgery + WBRT		P-value
	N	%	N	%		N	%	N	%		N	%	N	%	
	5158		5158			4355		4355			7655		7655		
Sex					1										
Male	3090	59.91	3090	59.91		2650	60.85	2650	60.85	1	4007	52.34	4007	52.34	1
Female	2068	40.09	2068	40.09		1705	39.15	1705	39.15		3648	47.66	3648	47.66	
Age	62.88±10.49		62.88±10.49		1	61.49±9.87		61.49±9.87		1	57.67±10.63	57.67±10.63	1		
20~29	8	0.16	8	0.16		3	0.07	3	0.07	1	26	0.34	26	0.34	1
30~39	122	2.37	122	2.37		96	2.2	96	2.2		389	5.08	389	5.08	
40~49	479	9.29	479	9.29		460	10.56	460	10.56		1307	17.07	1307	17.07	
50~59	1204	23.34	1204	23.34		1172	26.91	1172	26.91		2466	32.21	2466	32.21	
60~69	1755	34.02	1755	34.02		1596	36.65	1596	36.65		2342	30.59	2342	30.59	
70~79	1590	30.83	1590	30.83		1028	23.61	1028	23.61		1125	14.7	1125	14.7	
Medical aid beneficiary	228	4.42	216	4.19	0.5605	186	4.27	173	3.97	0.4835	444	5.8	345	4.51	0.0003
Medial facility															
Senior general hospital	2843	55.12	2869	55.62	0.8711	2490	57.18	2407	55.27	0.0023	3741	48.87	4032	52.67	<.0001
Tertiary hospital	614	11.9	604	11.71		482	11.07	423	9.71		819	10.7	686	8.96	
Secondary hospital	1701	32.98	1685	32.67		1383	31.76	1525	35.02		3095	40.43	2937	38.37	
Local clinic															
Primary cancer															
Head and neck	70	1.36	53	1.03	0.1231	63	1.45	54	1.24	0.4022	186	2.43	139	1.82	0.0084
Esophagus	40	0.78	24	0.47	0.0448	35	0.8	38	0.87	0.7244	87	1.14	73	0.95	0.2659
Stomach	202	3.92	173	3.35	0.1271	148	3.4	123	2.82	0.1229	204	2.66	174	2.27	0.1182
Colorectal	558	10.82	523	10.14	0.2605	472	10.84	491	11.27	0.5162	775	10.12	810	10.58	0.3531
Liver	272	5.27	238	4.61	0.1225	237	5.44	222	5.1	0.4719	477	6.23	389	5.08	0.0021
Hepatobiliary	45	0.87	40	0.78	0.586	28	0.64	30	0.69	0.7922	54	0.71	36	0.47	0.057
Pancreas	90	1.74	74	1.43	0.2079	60	1.38	57	1.31	0.7801	109	1.42	94	1.23	0.2892
Pharynx	17	0.33	11	0.21	0.2562	13	0.3	20	0.46	0.2221	34	0.44	32	0.42	0.8051
Lung	3585	69.5	3710	71.93	0.0068	3026	69.48	3153	72.4	0.0027	4491	58.67	4838	63.2	<.0001

Breast		302	5.85	335	6.49	0.1771	276	6.34	274	6.29	0.9298	1600	20.9	1426	18.63	0.0004
Cervix		28	0.54	23	0.45	0.4828	22	0.51	30	0.69	0.2658	113	1.48	94	1.23	0.1836
Uterine		17	0.33	14	0.27	0.5895	18	0.41	21	0.48	0.6302	60	0.78	50	0.65	0.3386
Ovary		92	1.78	79	1.53	0.3161	69	1.58	66	1.52	0.7947	94	1.23	106	1.38	0.393
Prostate		112	2.17	98	1.9	0.329	96	2.2	101	2.32	0.7186	170	2.22	156	2.04	0.4332
Scrotum		4	0.08	4	0.08	1	2	0.05	1	0.02	0.5636	3	0.04	6	0.08	0.3172
Kidney		255	4.94	198	3.84	0.0062	250	5.74	191	4.39	0.0039	208	2.72	240	3.14	0.1249
Bladder		57	1.11	37	0.72	0.0382	42	0.96	39	0.9	0.7377	61	0.8	50	0.65	0.2947
Thyroid		111	2.15	105	2.04	0.6799	99	2.27	99	2.27	1	147	1.92	173	2.26	0.1419
Operation		412	7.99	394	7.64	0.509	428	9.83	377	8.66	0.0592	585	7.64	848	11.08	<.0001
Chemotherapy		2763	53.57	2787	54.03	0.6355	2444	56.12	2492	57.22	0.2993	4766	62.26	4992	65.21	0.0001
Diagnostic year																
	2005	177	3.43	164	3.18	0.5496	156	3.58	140	3.21	0.8491	331	4.32	258	3.37	<.0001
	2006	242	4.69	248	4.81		206	4.73	196	4.5		472	6.17	359	4.69	
	2007	262	5.08	244	4.73		225	5.17	212	4.87		496	6.48	426	5.56	
	2008	346	6.71	347	6.73		313	7.19	287	6.59		603	7.88	527	6.88	
	2009	410	7.95	391	7.58		370	8.5	372	8.54		748	9.77	651	8.5	
	2010	438	8.49	459	8.9		382	8.77	382	8.77		628	8.2	703	9.18	
	2011	463	8.98	441	8.55		382	8.77	400	9.18		631	8.24	752	9.82	
	2012	413	8.01	392	7.6		366	8.4	395	9.07		633	8.27	730	9.54	
	2013	504	9.77	480	9.31		416	9.55	450	10.33		731	9.55	796	10.4	
	2014	560	10.86	543	10.53		494	11.34	500	11.48		695	9.08	829	10.83	
	2015	602	11.67	685	13.28		506	11.62	511	11.73		928	12.12	864	11.29	
	2016	741	14.37	764	14.81		539	12.38	510	11.71		759	9.92	760	9.93	
CCI		5.10±2.18		5.06±2.39		0.438	5.10±2.14		5.19±2.07		0.0318	4.09±2.35		5.07±1.99		<.0001

Abbreviations : RT: radio therapy, PSM: propensity score matching, CCI: Charlson Comorbidity Index, WBRT, whole-brain radiotherapy

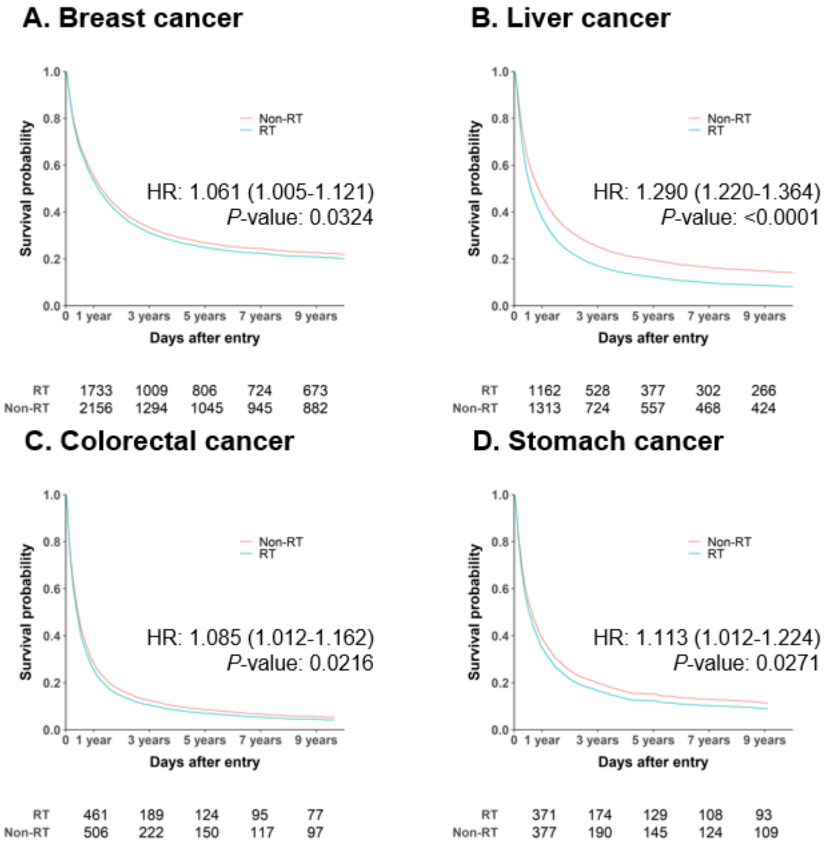

Supplemental figure 1. Cox regression survival analyses for breast cancer (A), liver cancer (B), colorectal cancer (C), and stomach cancer (D). HR: hazard ratio.

Abbreviations : Non-RT: Non-radio therapy, RT: radio therapy, HR: Hazard ratio,

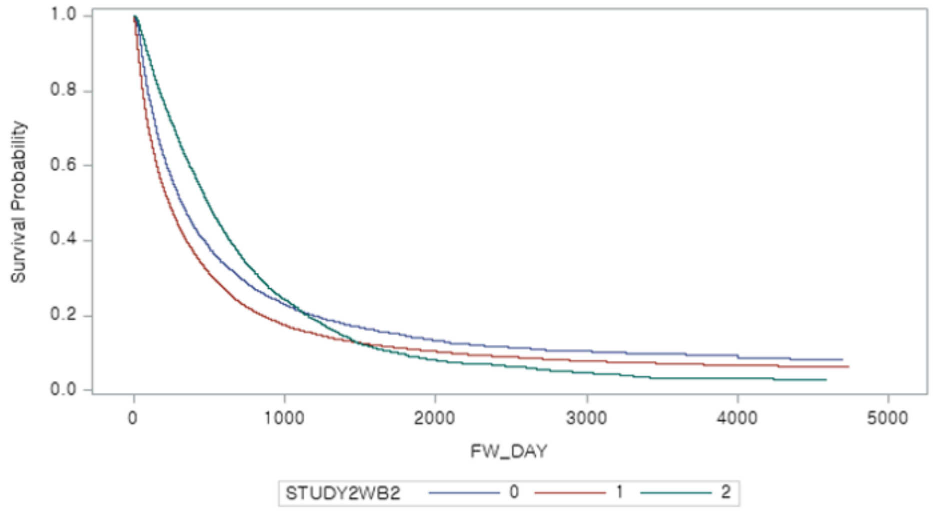

	Start	1year	3years	5years	7years	9years
**0 Radiosurgery**	**5276**	**2392**	**710**	**316**	**156**	**67**
**1 WBRT**	**42186**	**12954**	**4116**	**2269**	**1450**	**962**
**2 Radiosurgery + WBRT**	**7871**	**4322**	**1136**	**369**	**153**	**46**

Supplemental figure 2. Kaplan-Meier survival curves for Radiosurgery, WBRT, Radiosurgery+WBRT.

Abbreviations :WBRT: whole-brain radiotherapy

References

1. Charlson ME, Pompei P, Ales KL and MacKenzie CR. A new method of classifying prognostic comorbidity in longitudinal studies: development and validation. *Journal of chronic diseases*. 1987;40:373-83.
